# Recovering skin-nerve interaction by nanoscale metal-organic framework for diabetic ulcers healing

**DOI:** 10.1016/j.bioactmat.2024.08.024

**Published:** 2024-08-29

**Authors:** Xiuru Ji, Jingwei Zhou, Zengding Zhou, Zeyang Liu, Li Yan, Yuhan Li, Haiyan Guo, Weijie Su, Han Wang, Dalong Ni

**Affiliations:** a Department of Orthopaedics, Shanghai Key Laboratory for Prevention and Treatment of Bone and Joint Diseases, Shanghai Institute of Traumatology and Orthopaedics, Ruijin Hospital, Shanghai Jiao Tong University School of Medicine, No. 197, Ruijin 2nd Rd, Shanghai, 200025, PR China; b Department of Plastic and Reconstructive Surgery, Shanghai Ninth People's Hospital, Shanghai Jiao Tong University School of Medicine, No. 639, Zhizaoju Rd, Shanghai, 200011, PR China; c Department of Burn and Plastic Surgery, Ruijin Hospital, Shanghai Jiao Tong University School of Medicine, No. 197, Ruijin 2nd Rd, Shanghai, 200025, PR China; d Department of Dermatology, Ruijin Hospital, Shanghai Jiao Tong University School of Medicine, No. 197, Ruijin 2nd Rd, Shanghai, 200025, PR China

**Keywords:** Diabetic ulcers, Reactive oxygen species, Metal-organic framework, Cerium, Neuroendocrine

## Abstract

Skin-nerve interaction plays an important role in promoting wound healing. However, in diabetic ulcers (DUs), the diabetic periphery neuropathy and excessive levels of reactive oxygen species (ROS) block skin-nerve interaction and further impede the DUs healing. Herein, we developed a nanoscale metal-organic framework loaded with nerve growth factor (NGF/Ce-UiO-66, denoted NGF/CU) for the treatment of DUs. The Ce-UiO-66 (CU) was applied as an antioxidant to scavenge ROS and reduce the inflammatory response while the NGF aided in the recovery of cutaneous nerves to further promote DUs healing. Both *in vitro* and *in vivo* experiments revealed the effective ability of NGF/CU for DUs healing. Subsequent RNA sequencing analysis revealed the mechanism that NGF/CU can improve wound healing by inhibiting the NF-κB signaling pathway and recovering the neuroendocrine system of the skin. This strategy of nerve regulation will provide more ideas for the treatment of DUs and other organ injuries.

## Highlights

• A nanoscale metal-organic framework loaded with nerve growth factor (NGF/Ce-UiO-66, denoted NGF/CU) was developed for diabetic ulcers (DUs) treatment via ROS scavenging and cutaneous nerve recovering.
• NGF/CU can improve wound healing by inhibiting the NF-κB signaling pathway and recovering the neuroendocrine system of the skin.
• New insights into the interaction between skin and nerves for the treatment of DUs.

## Graphical abstract

**Figure:**
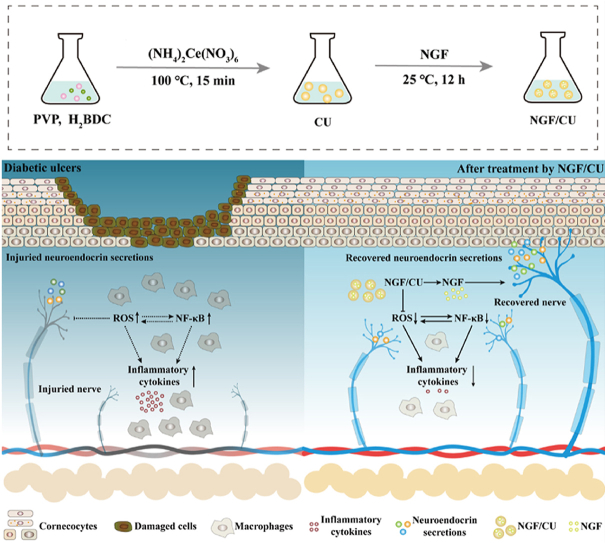

## 1 Introduction

Diabetic ulcers (DUs), a common and serious complication of diabetes, appear as open sores usually on the feet and legs. The DUs usually present a long-term non-healing state, which makes them difficult to be cured [1]. Current clinical treatments for DUs are mainly focused on regular local thorough debridement as well as enhanced metabolic management [2]. However, up to 50 % of patients still experience clinical recurrence, and approximately 20 % of patients are threatened with amputation. The pathogenesis of DUs is complex and partly indeterminate [3]. But generally, diabetes will induce systemic metabolic disorders, leading to impaired normal functions of various organs and tissues, such as skin, nerves, kidneys, eyes, and cerebral vessels [4]. Hence, when DUs occur, the skin tissues can hardly be repaired by themselves in time, resulting in a long-term inflammatory state and related reactive oxygen species (ROS) accumulation in the wound. In turn, the overexpressed ROS will further aggravate inflammation by damaging normal cells and convening proinflammatory cytokines [5]. The vicious circle seriously impairs the local homeostasis of the skin, resulting in a slow and broken wound-healing process. Therefore, it is imperative to develop effective treatment strategies to break this vicious circle for DUs.

Recent progress has revealed that nerves play important roles in regulating and maintaining the homeostasis of certain organs, such as the intestine and skin [6]. For instance, the cutaneous nerves can regulate skin homeostasis via the neuroendocrine system [7]. Some skin cells (e.g., skin Merkel cells [8]), nervous centralis (e.g., hypothalamus, hypophysis), and endocrine cells (e.g., adrenal gland) have been proven to participate in nerve-skin regulation b), [9]. The neuroendocrine system can exhibit an anti-inflammatory effect and promote the repair of skin injury by secreting cortisol and various neuropeptides (e.g., substance P, adrenocorticotropic hormone) [10]. However, diabetes will impair the normal function of cutaneous nerves [11]. It is reported that the expression level of nerve growth factor (NGF), a crucial factor in neuronal maintenance and survival, was significantly reduced in diabetes patients [12]. In fact, exogenous NGF supplementation has been demonstrated to improve cutaneous nerve regeneration and wound healing [3,13]. In addition, the high-level ROS in DUs will further damage cutaneous nerves a), [11]. Hence, delivering NGF and scavenging ROS simultaneously have much potential to recover cutaneous nerve function and promote the repair of DUs.

In the past decade, metal-organic frameworks (MOFs) have drawn numerous attention because of their rich performance and distinctive properties [14]. Especially, due to their adjustable composition, structural versatility, and biocompatibility, nanoscale MOFs have been extensively used in the area of nanomedicine, such as drug delivery, scavenging ROS, fluorescence imaging, and radiosensitization [15]. Herein, NGF-loaded Ce-UiO-66 (NGF/CU) was investigated for DUs treatment. The MOFs of Ce-UiO-66 (CU) can scavenge overproduced ROS and regulate inflammatory responses in both the diabetic peripheral neuropathy and the wound microenvironment [16]. Importantly, the NGF stored in the frameworks of CU is used as a supplement to promote the recovery of cutaneous nerve and related function. Both *in vitro* and *in vivo* experiments showed that NGF/CU could effectively facilitate the healing progress of DUs. The RNA-sequence analysis further revealed the underlying mechanism of NGF/CU promoting DUs healing, which was through blocking the NF-κB signaling pathway for anti-inflammation and activating the neuroendocrine system for skin-regeneration promotion. We believe that this study will provide new insights into the treatments of DUs.

## 2 Results and discussion

### 2.1 Synthesis and characterization of Ce-UiO-66 and NGF/Ce-UiO-66

**Fig. 1:**
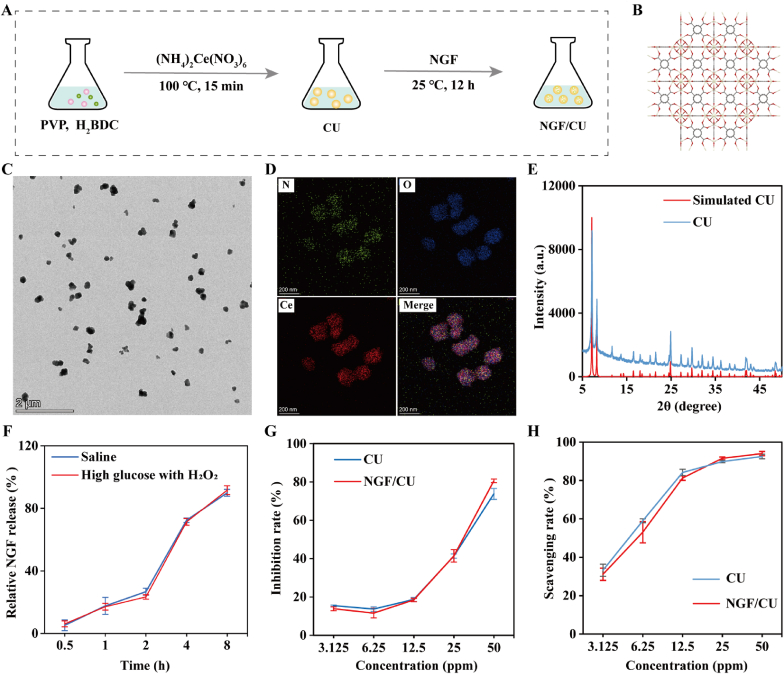
Synthesis and characterization of NGF/CU. (A) Schematic illustration of the synthesis progress of NGF/CU. (B) Crystal structure, (C) TEM image, (D) Elemental mapping, and (E) XRD analysis of CU. (F) The release of NGF from NGF/CU (n = 3, mean ± SD). (G) The total antioxidant capacity of NGF/CU (n = 3, mean ± SD). (H) The ROS scavenging ability of NGF/CU (n = 3, mean ± SD).

The NGF/CU was synthesized via a conventional hydrothermal technique as shown in Fig. 1A. The size of CU was around 200 nm as demonstrated by transmission electron microscopy (TEM) in Fig. 1C. Elemental mapping results exhibited the existence of nitrogen, oxygen, and cerium in CU (Fig. 1D). X-ray diffraction (XRD) analysis revealed that the structure of CU was in accordance with that of Ce_6_O_4_(OH)_4_(BDC)_6_ (Fig. 1E), and the structure of CU was illustrated in Fig. 1B [17]. Fourier transform infrared (FT-IR) spectroscopy of CU was shown in Fig. S1 with an absorption peak near 1633 cm^−1^, indicating the presence of the carbonyl group (C=O), which was due to the involvement of 1,4-benzenedicarboxylic acid (H_2_BDC). As shown in Fig. S2, the residual mass (i.e., ceria) of CU was around 44 % according to the thermogravimetric analysis, which demonstrated the content of Ce was approximately 35.6 % in CU. Considering the microenvironment of DUs, a high glucose and ROS system containing 16.7 mM glucose and 100 μM H_2_O_2_ was established [1b]. Then the release experiment was performed in the saline and high glucose and ROS system, respectively. As shown in Fig. 1F, there were no significant differences in the release profiles between the saline and high glucose and ROS system. The NGF was gradually released from NGF/CU in both saline and high glucose and ROS system, confirming the presence and gradual effective release of NGF over time from NGF/CU (Fig. 1F). According to dynamic light scattering (DLS) studies, the average hydrodynamic diameters of CU and NGF/CU were approximately 200 nm (Figs. S3 and S4), which was consistent with the TEM data, verifying that the addition of NGF did not influence the size of CU. These above results demonstrated the successful synthesis of NGF/CU.

CU exhibits catalytic and photocatalytic characteristics as a result of the existence of Ce(III)/Ce(IV) redox pairs, which enable it to scavenge reactive oxygen species (ROS) through its potent antioxidant activity. Previous studies have confirmed that Ce^3+^/Ce^4+^ electron pairs could generate oxygen through the reaction with ROS. Ce^3+^ is capable of triggering the conversion of superoxide anion to H_2_O_2_, and Ce^4+^ could oxidize H_2_O_2_ into water and oxygen. This process continues cyclically, allowing CU to efficiently remove ROS and display strong antioxidant properties [16,18]. The antioxidant ability of NGF/CU was further investigated in the solution. The total antioxidant capacity of NGF/CU was firstly detected. As shown in Fig. 1G, both CU and NGF/CU exhibited remarkable antioxidant ability. When the concentration of CU and NGF/CU was up to 50 ppm, nearly 80 % oxidation reaction could be inhibited. Then the ability of CU and NGF/CU to scavenge ROS such as H_2_O_2_ was evaluated. As shown in Fig. 1H and 25 ppm of CU and NGF/CU decomposed around 90 % of total H_2_O_2_ in the solution. These results indicated that the NGF/CU had effective antioxidant capacity and the addition of NGF did not influence the antioxidant ability of CU.

### 2.2 NGF/CU protect skin cells from ROS damage

**Fig. 2:**
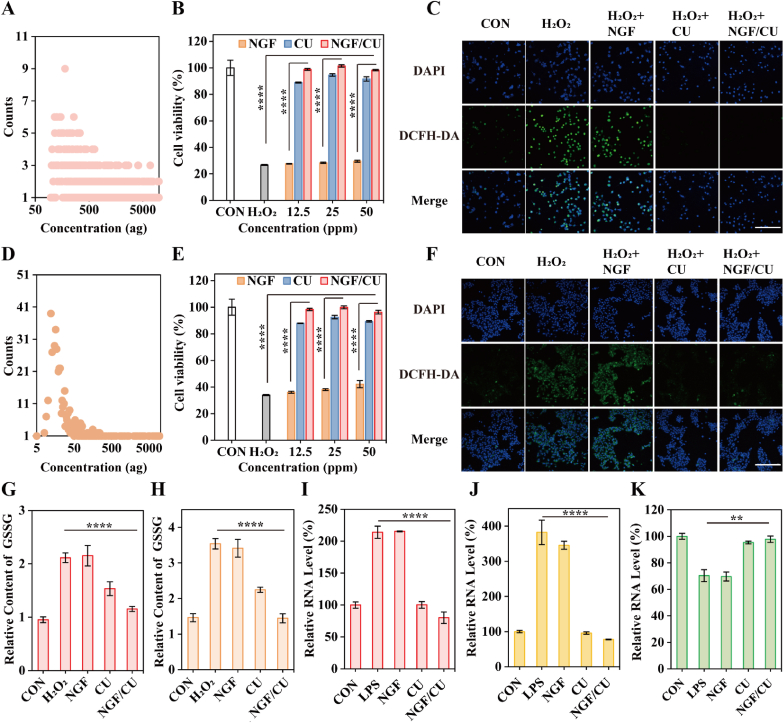
NGF/CU protects cells from ROS damage. Cytophagy of NGF/CU by (A) HUVECs and (D) HaCaTs. Cell viability of H_2_O_2_-stimulated (B) HUVECs and (E) HaCaTs with different concentrations of NGF/CU (n = 3, mean ± SD). ROS detection of H_2_O_2_-stimulated (C) HUVECs and (F) HaCaTs with different treatments by DCFH-DA staining. GSSG detection of H_2_O_2_-stimulated (G) HUVECs and (H) HaCaTs in each group (n = 3, mean ± SD). Expression level of relative inflammation cytokines including (I) TNF-α, (J) IL-6, and (K) IL-10 in LPS-stimulated RAW264.7 cells line in each group (n = 3, mean ± SD). Scale bar: 100 μm ** indicating 0.001< p < 0.01, **** indicating p < 0.0001 according to ANOVA analysis.

Multiple skin cellular behaviors play important roles in the wound healing progress, such as endothelial cell differentiation and keratinocyte migration [19]. Thus, we investigated the efficacy of NGF/CU that protected human umbilical vein endothelial cells (HUVECs) and human keratinocyte cells (HaCaTs) from ROS damage. First, cellular uptake of NGF/CU was measured through single cell-inductively coupled plasma-mass spectrometry (SC-ICP-MS) technology. The diagrams presented in Fig. 2A and D indicated that the cells were proficient in internalizing NGF/CU. The mean values of accumulation of Ce were 3954 ag/cell and 1352 ag/cell (1 ag = 10^−18^ g) and median values of that were 1857 ag/cell and 26 ag/cell in the HUVECs and HaCaTs, respectively. The cell viability of cells with H_2_O_2_ (100 μM) incubation under different treatments was also assessed. In Fig. 2B and E, the cell viability of H_2_O_2_-treated HUVECs and HaCaTs were both about 30 %, which was similar to cells treated with H_2_O_2_ and NGF together, while the viability of H_2_O_2_-stimulated cells treated with CU and NGF/CU were nearly recovered to 100 %, showing the favorable H_2_O_2_ scavenging ability of NGF/CU. The potential competence of NGF/CU in countering ROS was then evaluated *in vitro* by DCFH-DA staining, a fluorescent dye known to be sensitive to ROS [5d]. As shown in Fig. 2C and F, the intracellular green fluorescent signal of both HUVECs and HaCaTs increased dramatically after stimulation with H_2_O_2_ compared to the control group without H_2_O_2_ treatment. Conversely, the intracellular ROS level noticeably decreased after the application of CU and NGF/CU in both HUVECs and HaCaTs. Importantly, the content of oxidized glutathione (GSSG) markedly rose upon H_2_O_2_ treatment but decreased to a normal level after treatment with CU and NGF/CU in both HUVECs and HaCaTs compared to the control group (Fig. 2G and H), indicating a shift in inflammatory levels within cells. These data indicated that NGF/CU could protect wound-healing-related cells from ROS damage and regulate the inflammatory state of wound microenvironment.

It is commonly known that excess ROS in the DUs can trigger a severe inflammatory response, causing changes in inflammatory cytokines such as tumor necrosis factor-α (TNF-α), interleukin-6 (IL-6), and interleukin-10 (IL-10) a), d). Hence, the expression of these inflammatory cytokines was further detected after scavenging ROS with NGF/CU. Lipopolysaccharides (LPS)-stimulated macrophages of RAW264.7 cells were used as inflammation model. ELISA results showed that the level of pro-inflammatory factors (TNF-α and IL-6) was extremely increased in LPS-stimulated RAW264.7 cells, while the content of TNF-α and IL-6 was extremely decreased by CU and NGF/CU (Fig. 2I-K). Furthermore, the expression of anti-inflammatory factors IL-10 slightly decreased due to LPS stimulation, but CU and NGF/CU treatment could recover its expression to the normal level. The anti-inflammatory characteristics of NGF/CU were also evidenced by the decreased fluorescence of TNF-α and IL-6 in the LPS-stimulated RAW264.7 cells treated with CU and NGF/CU, and that of IL-10 presented an opposite outcome (Figs. S5a–c). In conclusion, NGF/CU has the ability to eliminate high-level ROS and decrease severe inflammatory responses. Notably, whether it was the regulation of intracellular ROS or inflammatory cytokines, the single NGF group did not show obvious significant antioxidant capacity, indicating that CU was the main antioxidant agent in NGF/CU.

### 2.3 NGF/CU repair the injury of nerve cells

**Fig. 3:**
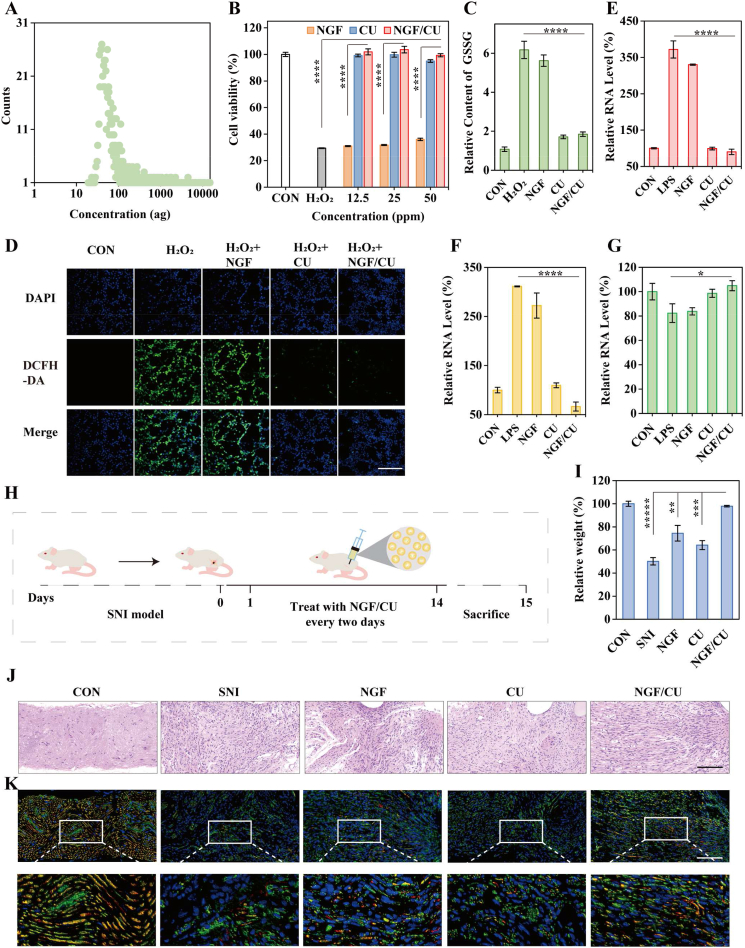
NGF/CU repair periphery neuropathy. (A) Cytophagy of NGF/CU by PC12 cells. (B) Cell viability of H_2_O_2_-stimulated PC12 cells with different concentrations of NGF/CU (n = 3, mean ± SD). (C) GSSG detection of H_2_O_2_-stimulated PC12 cells under different treatment conditions (n = 3, mean ± SD). (D) ROS detection of H_2_O_2_-stimulated PC12 cells in each group by DCFH-DA staining. Expression level of relative inflammation cytokines including (E) TNF-α, (F) IL-6, and (G) IL-10 in LPS-stimulated BV2 cells line (n = 3, mean ± SD). (H) Schematic illustration of the establishment of the SNI model. (I) Weight of calf muscles from mice with different treatments. (J) H&E staining of the injured sciatic nerve with different treatments. (K) Immunofluorescence stain of NF200 (Red) and Tuj1 (Green) in each group. Scale bar: 100 μm * indicating 0.01< p < 0.05, ** indicating 0.001< p < 0.01, *** indicating 0.0001< p < 0.001, **** indicating p < 0.0001 according to ANOVA analysis.

The cutaneous nerve system is critical in facilitating wound healing. However, patients with diabetic milieu have severe diabetic periphery neuropathy, which is the main cause of DUs and significantly impedes the healing of diabetic ulcers [3]. Recent research has indicated that high levels of ROS and the lack of NGF in diabetic patients contribute to the onset of diabetic peripheral neuropathy [11a]. Therefore, scavenging ROS and supplying NGF are essential for recovering cutaneous neuropathy to promote DUs healing. We initially examined the cytophagy of NGF/CU on PC12 cells, which revealed an average Ce content of approximately 955 ag/cell (Fig. 3A). Next, the ROS scavenging ability of NGF/CU was assessed on H_2_O_2_-stimulated PC12 cells. The Cell Counting Kit-8 (CCK-8) results demonstrated that CU and NGF/CU could improve the cell viability of H_2_O_2_-stimulated PC12 cells (Fig. 3B). What's more, after treatment with CU and NGF/CU, the cellular GSSG levels returned to normal, indicating a reduction of oxidative stress levels in cells (Fig. 3C), in accordance with the result of *in vitro* ROS detection (Fig. 3D). The ability of NGF/CU to regulate inflammation cytokines on microglia-BV2 cells was also examined. The increased fluorescence of TNF-α and IL-6 on LPS-stimulated BV2 cells (Figs. S6a–b) was observed but decreased rapidly after being treated with CU and NGF/CU, and IL-10 presented an opposite outcome (Fig. S6c). Results obtained from ELISA kits also validated these findings, which aligned with the results from immunofluorescence (Fig. 3E-G). These results demonstrated that NGF/CU could relieve the inflammation and oxidative stress state of neural cells.

The spared nerve injury (SNI) model was further established to detect reparative effects of NGF/CU on the nerve cells *in vivo*. The design and treatment process of the SNI model are illustrated in Fig. 3H. After 14 days of treatment, we took the damaged nerves and calf muscles of two sides from the mice for further evaluation. As shown in Fig. S7, in the SNI, NGF, and CU groups, the calf muscles of the nerve-damaged side were much smaller than the undamaged side, while the muscles in the mice treated with NGF/CU were comparable to those in the control group without nerve damaging. The relative calf muscle mass in the SNI, NGF, CU, and NGF/CU groups were around 50 %, 74 %, 64 %, and 98 %, respectively, indicating that calf muscle mass in NGF/CU-treated mice showed no significant atrophy compared to the control group (Fig. 3I). The damaged nerve conditions following different treatments were also detected. In contrast to the control group, H&E staining revealed that nerve tissues in the SNI group were highly inflamed and disorganized, while the structure was more organized and the inflammation was lower in the NGF/CU group (Fig. 3J). In addition, the nerve structure was more disorganized and inflammatory activity was lower in the CU group than those in the NGF group. Neurofilament 200 (NF200) and β-III-tubulin (Tuj1) were two important indicators for the regeneration of periphery nerves [20]. The immunofluorescence assay of NF200 (Red) and Tuj1 (Green) were detected to assess the effectiveness of NGF/CU in promoting nerve regeneration. As shown in Fig. 3K and Fig. S8, in comparison to the SNI group, the NGF/CU group demonstrated substantial nerve regeneration, while the NGF group showed some improvement and the CU group had the lowest level of recovery, indicating the crucial function of NGF in promoting nerve repair. These findings indicated that CU could modulate the inflammatory state, but only in combination with NGF could it effectively alleviate the injury of the nerve.

### 2.4 Repaired nerves facilitate skin cell proliferation

**Fig. 4:**
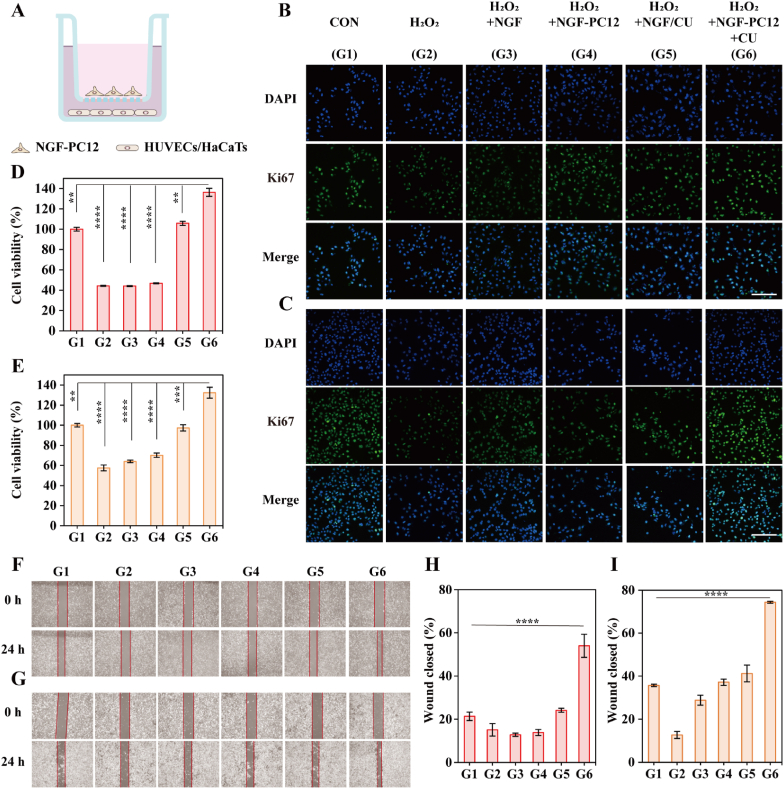
NGF/CU promoted cell proliferation via periphery nerve regeneration. (A) Schematic illustration of HUVECs and HaCaTs incubation with NGF-PC12 cells. Ki67 immunofluorescence of H_2_O_2_-stimulated (B) HUVECs and (C) HaCaTs with different treatments. Cell viability of H_2_O_2_-stimulated (D) HUVECs and (E) HaCaTs with different treatments (n = 3, mean ± SD). Cell scratch analysis of (F) HUVECs and (G) HaCaTs under different treatments. Quantitative analysis of cell scratch analysis for (H) HUVECs and (I) HaCaTs. Scale bar: 100 μm **** indicating p < 0.0001 according to ANOVA analysis.

Encouraged by the favorable function of NGF/CU in repairing periphery neuropathy, we then discovered the promoting potential of the periphery neural system in wound healing *in vitro*. A cell co-culture system was established using a transwell device, in which the NGF-treated PC12 cells (NGF-PC12) were put in the upper chamber while HUVECs or HaCaTs in the lower chamber (Fig. 4A). Herein, NGF was first incubated with PC12 cells to stimulate nerve functions of the cells. The skin cells were cultured with H_2_O_2_ for 6 h to establish inflammation models *in vitro* and then co-incubated with different treatments. As an indicator for cell proliferation activity, Ki67 was employed to assess the proliferation activity of HUVECs and HaCaTs. As shown in Fig. 4B and C, the presence of H_2_O_2_ significantly reduced skin cell proliferation activity compared with the control group, which was restored to the normal level after incubating with NGF/CU. In conditions of adding NGF-PC12 cells and CU together, the activity of skin cells increased significantly compared to the control group. These results were further confirmed by the qualitative analysis of Ki67 immunofluorescence intensity in Figs. S9a–b. Then cellular proliferation of skin cells was further investigated by a CCK-8 assay. As illustrated in Fig. 4D and E, the skin cell growth activity was inhibited by H_2_O_2_, which was recovered to the normal level after incubation with NGF/CU. Importantly, the growth rate of skin cells increased by nearly 40 % for adding NGF-PC12 with CU as compared with the control group, indicating the important roles of nerve cells in enhancing the proliferation of skin cells.

Furthermore, cellular scratch experiments were utilized to simulate the process of wound healing (Fig. 4F-I). It was easy to find that the closure rate of cells incubated with NGF-PC12 nerve cells with CU together was much higher than that of other different treatment groups. The excessive production of ROS could be effectively eliminated by NGF/CU, which significantly decreased the inhibitory effect of ROS on skin cell growth. However, the closure rate of skin cells was not notably enhanced unless incubated with NGF-PC12 nerve cells and CU together. These findings suggested that nerve cells are essential in the proliferation of skin cells. Therefore, we suppose that some substances secreted from healthy nerve cells may assist the process of wound healing, while damaged nerve cells cannot secrete enough substances. The species of these substances will be verified in the following experiments.

### 2.5 NGF/CU accelerate wound healing *in vivo*

**Fig. 5:**
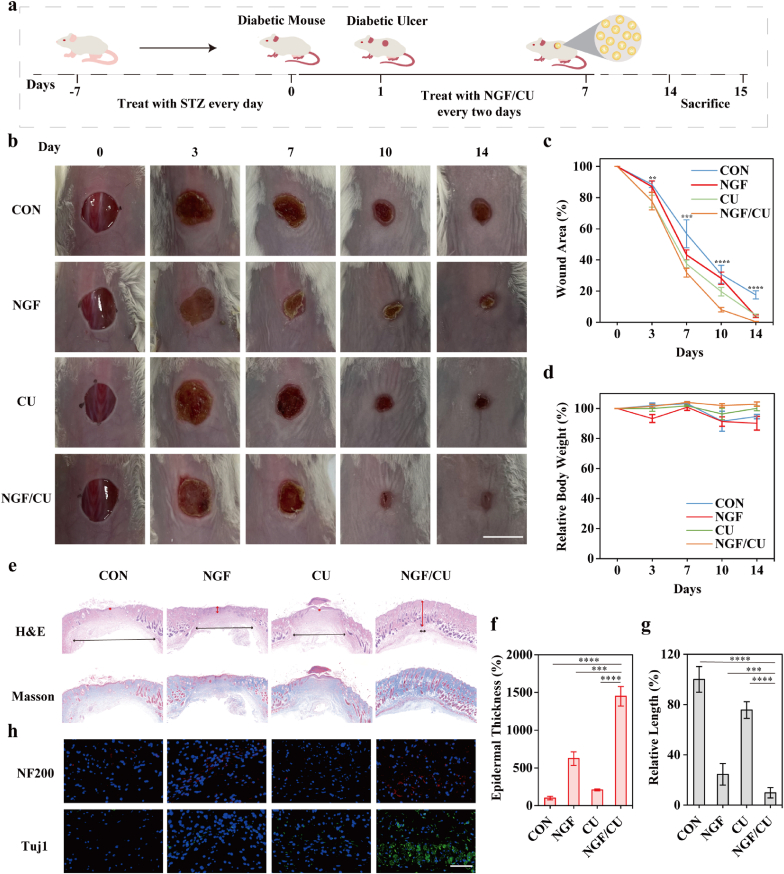
NGF/CU accelerated DUs healing. (a) Schematic illustration of the establishment of the DUs model. (b) Representative photographic images of the DUs wounds with different treatments on 0, 3, 7, 10, and 14 days, respectively (Scale bar: 1 cm). (c) Quantification of the wound area rate in each group (n = 5, mean ± SD). (d) Weight of diabetic mice with different treatments on 0, 3, 7, 10, and 14 days, respectively (n = 5, mean ± SD). (e) H&E and Masson Trichrome staining of wounds after different treatments. (f) Quantification of epidermal thickness in each group (n = 3, mean ± SD). (g) Quantification of wound length in each group (n = 3, mean ± SD). (h) Immunofluorescence stain of NF200 and Tuj1 in each group (Scale bar: 100 μm). ** indicating 0.001< p < 0.01, *** indicating 0.0001< p < 0.001, **** indicating p < 0.0001 according to ANOVA analysis.

To determine the effects of NGF/CU on promoting DUs healing, we constructed wounds on diabetic mice and evaluated the wound healing quality. As illustrated in Fig. 5a, diabetic wounds were performed on the back of the mice with streptozotocin (STZ)-induced diabetes. These mice were randomly divided into the control (treated with Saline), NGF, CU, and NGF/CU groups. The sizes and photographs of the wound areas in different treatment groups were collected on days 0, 3, 7, 10, and 14. As shown in Fig. 5b and c, NGF/CU possessed excellent wound-healing ability. Quantitatively, the wound closure of the NGF/CU group was about 68 %, 91 %, and 99 % on days 7, 10, and 14, respectively, which was higher than the control group (43 %, 69 %, and 82 %, respectively). It was worth mentioning that mice treated with the NGF or CU only group also showed a good closure rate, but the healing rate was lower than that of NGF/CU group. The body weights of mice were also collected, where no significant changes were found in the NGF/CU group (Fig. 5d).

The progress of cutaneous nerve regeneration was also evaluated. NF200 and Tuj1 were employed as indicators for peripheral nerve regeneration through immunofluorescence assays and quantitative reverse transcription polymerase chain reaction (RT-qPCR) analysis. As shown in Figs. S10a–b, the expression of the two indicators gradually increased over time in different treatment groups, correlating with the quality of wound healing (Fig. 5b and c). The group treated with NGF/CU exhibited better peripheral nerve regeneration at the wound site (red arrows), resulting in better wound healing outcomes compared to the control group. It was also worth pointing that the DUs healing progress in CU group was much lower than NGF/CU. This would be related to the poor cutaneous nerve regeneration in CU group, highlighting the function of cutaneous nerve in regulating wound healing and skin homeostasis. Moreover, the recovered cutaneous nerve system quality in the NGF group was quite well but lower than that in the NGF/CU group, illustrating that scavenging ROS and reducing the inflammatory response exerted a positive effect on neural and wound repair. Therefore, these results emphasized the re-innervation of the cutaneous nerve system in DUs healing progress. The supplementary of NGF combined with the reduction of inflammatory response in the wound site promotes the recovery of the cutaneous nerve system, thereby regulating the DUs microenvironment and accelerating wound healing.

After 14 days, the wounds samples from each treatment group were gathered and analyzed by histological analysis, including H&E staining and Masson Trichrome staining. As depicted in Fig. 5e, better ordered epithelial tissues and more clearly defined granulation tissues were shown in the NGF/CU group compared to the other groups in the H&E staining. Masson Trichrome staining revealed that in the NGF/CU groups, collagen protein was arranged neatly and occupied a considerably greater volume, substantiating their ability to promote extracellular matrix formation. Quantitative analysis of epidermal thickness indicated that NGF/CU had better effect on the repair of epidermal tissue (Fig. 5f). The wound length in the NGF/CU group was smaller than that in other treatment groups, indicating the good regeneration of the skin tissues (Fig. 5g). More importantly, although the wound area in NGF group was larger than CU group, the recovery quality of NGF group was superior to the control and CU groups, which could be attributed to the regulatory effect of repaired nerves on the skin, thereby accelerating DUs healing.

Blood vessels are particularly crucial in the skin system in conveying a variety of substances, such as oxygen, growth factors, and nutrients. Insufficient blood supply to the affected area could impede successful wound healing [1b]. Hence, the development of new blood vessels is efficient for the restoration of the skin system. The blood vessels after treatment in different groups were then evaluated by ultrasound and photoacoustic imaging systems. As shown in Fig. S11, the blood vessels gradually increased with time and the NGF/CU conveyed the most blood vessels compared to other groups. Meanwhile, as specific markers for capillaries, the CD31 and α-SMA were selected for neovascularization identification [21]. The new generation of microvessels in the NGF/CU group was extremely higher than that in other treatment groups, especially in the control group (Fig. S12). These results showed that NGF/CU could notably accelerate blood vessel formation and promote DUs healing.

Finally, we assessed the restored quality of the cutaneous nerves in the skin. According to the immunofluorescence assay of NF200 and Tuj1 shown in Fig. 5h and Fig. S13, the NGF/CU group had the strongest fluorescence, followed by the NGF group, and the CU group was only marginally higher than the control group, suggesting the significant function of cutaneous nerve in the skin homeostasis and wound healing. These findings revealed that NGF/CU successfully scavenged ROS and reversed severe cutaneous neuropathy to improve DUs repair.

### 2.6 Mechanism of NGF/CU promoting DUs healing

Although peripheral nerves were implicated in both the development and resolution of DUs, the mechanisms behind this association remain unclear. Hence, skin tissues from mice receiving various treatments (Saline, NGF, CU, NGF/CU) were collected for RNA sequencing analysis to investigate probable mechanisms. Different transcriptome profiles were found for the control, NGF, CU, and NGF/CU groups using principal component analysis (PCA) (Fig. S14a). Comparing the control group to those treated with NGF, CU, or NGF/CU, volcano plots showed that there were 55, 89, and 66 upregulated genes, while 833, 1044, and 943 downregulated genes, respectively (Fig. S14c). Among these genes, 53 and 244 genes were shared between each pair of comparisons (NGF *Vs* CON and NGF/CU *Vs* CON, CU *Vs* CON and NGF/CU *Vs* CON, respectively), which represented the different functions of NGF and CU in the treatment of DUs (Fig. S14b).

**Fig. 6:**
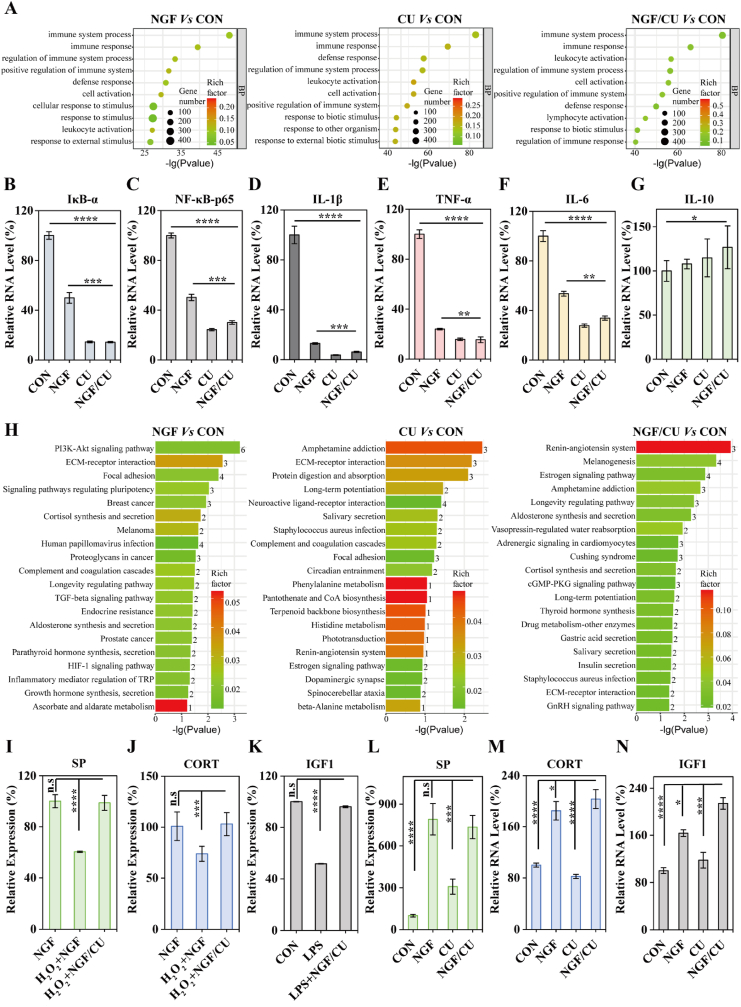
Mechanism of NGF/CU promoting DUs healing. (A) The GO pathway enriched downregulated genes for DUs with different treatments. Cutaneous mRNA levels of (B) IκB-α, (C) NF-κB-p65, (D) IL-1β, (E) TNF-α, (F) IL-6, and (G) IL-10. (H) The KEGG pathway enriched upregulated genes for DUs with different treatments. Expression of (I) SP, (J) CORT, and (K) IGF1 in nerve-related cells. Cutaneous mRNA levels of (L) SP and (N) IGF1. (M) Cutaneous expression of CORT. (n = 3, mean ± SD). * indicating 0.01< p < 0.05, ** indicating 0.001< p < 0.01, *** indicating 0.0001< p < 0.001, **** indicating p < 0.0001 according to ANOVA analysis.

The normalized heatmap revealed that following treatment with NGF/CU, the genes that responded to ROS were substantially reduced in diabetic ulcer mice (Fig. S14d). Furthermore, the antioxidant ability of NGF/CU was supported by a gene ontology (GO) pathway enrichment analysis, which demonstrated that the most ranked downregulated signaling pathways across all three comparison groups were predominantly linked to ROS (Fig. 6A). Surprisingly, the NGF group also exhibited certain anti-inflammatory effects, which would be related to the regulatory ability of repaired cutaneous nerve on the skin. The TNF signaling pathway, chemokine signaling pathway, and NF-κB signaling pathway were shown to be significantly enriched in relation to the anti-inflammatory mechanisms of the NGF/CU according to the KEGG (i.e., Kyoto Encyclopedia of Genes and Genomes) pathway enrichment analysis (Fig. S15). Studies have suggested that ROS would increase the production of inflammation cytokines, including TNF-α, IL-1β, and IL-6. Excessive inflammation in DUs could be caused by TNF-α triggering a severe inflammation along the TNF-α/NF-κB signaling pathways. Consistently, our findings suggested that NGF/CU blocked the NF-κB signaling pathway, thus lowering the expression of ROS and thereby reducing inflammation in DUs.

Then the capability of NGF/CU to scavenge ROS in DUs through the NF-κB signaling pathway was further investigated. RT-qPCR was performed to explore the effects of NGF/CU on the related proteins of the NF-κB signaling pathway, including IκB-α and NF-κB-p65. The expression of IκB-α and NF-κB-p65 was considerably reduced by NGF/CU compared to the control group, indicating that the NF-κB signaling pathway could be inhibited by NGF/CU (Fig. 6B and C). The mRNA expression of proinflammatory cytokines such as IL-6, IL-1β, TNF-α, and IL-10, which are related to the pathologies of DUs, was evaluated (Fig. 6D-G). These results revealed that pro-inflammation cytokines (i.e., IL-6, IL-1β, and TNF-α) dropped rapidly in DUs after being treated with NGF/CU, while the IL-10 increased slightly because of its anti-inflammatory capabilities, which were consistent with *in vitro* results. Furthermore, CU demonstrated a similar capability in the NF-κB signaling pathway inhibition and proinflammation cytokines regulation. The NF-κB signaling pathway was modestly repressed and the expression of proinflammation cytokines was lowered in the NGF group, which was associated with the recovery of the cutaneous nerve.

The NGF/CU had little influence on the proliferation of HUVECs and HaCaTs in cell line tests. However, they demonstrated substantial growth ability when incubated with nerve cells. This suggests that merely eliminating inflammation is insufficient for DUs repair, while the participation of the peripheral neural system is required to promote wound healing. From the GO and KEGG pathway upregulated enrichment analysis (Fig. 6H and Fig. S16), the elevated signaling pathways differed greatly amongst the three comparison groups. The top-ranking elevated signaling pathways in mice treated with NGF/CU were mostly related to the neuroendocrine system, such as cortisol synthesis and secretion, insulin secretion, and thyroid hormone synthesis, which were likewise increased in the NGF groups compared to the control group. However, no neuroendocrine-related pathways appeared in the top ranking of the CU group. These results demonstrated that the repaired cutaneous nerve could promote wound healing through the skin neuroendocrine. The elevated genes of response to the neuroendocrine were also considerably enriched in the NGF group and NGF/CU group, according to KEGG pathway enrichment analysis (Fig. 6H and Fig. S14d). Importantly, these genes were raised less in the NGF group than in the NGF/CU group while there was no discernible change in the CU group.

The skin is recognized as a neuroendocrine organ that produces several neuroendocrine secretions such as substance P (SP), cortisol (CORT), and insulin growth factor 1 (IGF1) a), b). SP is a neuropeptide produced by numerous cells [6d]. Its decreased expression in diabetic neuropathy can lead to disruption of skin tissues. Additionally, it has the potential to facilitate the migration of keratocytes, thereby accelerating wound healing and maintaining skin homeostasis [22]. As both well-known neuroendocrine secretions, CORT and IGF1 play a vital role in regulating local and systemic skin homeostasis b), [23]. IGF-1 has been extensively studied and proven to effectively suppress inflammatory responses and promote tissue repair, possibly through the activation of the PI3K-AKT signaling pathway [24]. This was consistent with the KEGG pathway upregulated analysis, which indicated that NGF/CU could promote wound healing through the PI3K-AKT signaling pathway [25]. Therefore, we tested these representative neuroendocrine chemicals *in vitro*. As shown in Fig. 6I-K, the content of SP, CORT, and IGF1 in nerve-related cells was decreased upon inflammatory stimulation, which recovered to the normal level after NGF/CU treatment. Then the related neuroendocrine chemicals *in vivo* were detected through ELISA kits and qPCR analysis. The results declared that SP, CORT, and IGF1 all increased after NGF/CU treatment compared to the DUs group without any treatment (Fig. 6L-N). More importantly, there was no noticeable difference between NGF and NGF/CU groups, indicating that the neuroendocrine system was mainly regulated by the supplement of NGF. These results confirmed our hypothesis regarding the promotion of skin cell growth by nerve cells via neuroendocrine secretions. The healing process of DUs was expedited through the restoration of cutaneous nerves via regulating neuroendocrine system. Therefore, NGF/CU could be employed for DUs treatment by inhibiting the NF-κB signaling pathway and regulating the neuroendocrine system, where CU was mainly responsible for ROS clearance and NF-κB signal pathway inhibition for anti-inflammation, and NGF mainly focused on cutaneous nerve repair and neuroendocrine regulation to maintain skin homeostasis and accelerate DUs healing.

### 2.7 Biocompatibility assessment of NGF/CU

Encouraged by the excellent DUs healing-promoting ability of NGF/CU, we proceeded to assess its biocompatibility. Firstly, HUVECs, HaCaTs, and PC12 cells were co-cultured with different concentrations of CU and NGF/CU for 24 h and then detected with CCK-8 assay kits. As illustrated in Figs. S17a–c, NGF/CU did not exhibit noticeable cytotoxicity at test concentrations. Additionally, we conducted the hemolysis experiments of NGF/CU and Triton-X-100 (TX-100) was used as the positive control. There was no apparent hemolysis observed compared to the TX-100 and Saline group, showing favorable biocompatibility of NGF/CU (Fig. S18). The biological safety of NGF/CU was performed *in vivo* with intradermal injection. After 0, 7 and 14 days treatment, the mice were sacrificed, and the major organs (skin, heart, liver, spleen, lung and kidney) were collected for further histological examination. As shown in Fig. S19a, hematoxylin and eosin (H&E) staining analysis revealed no evident detrimental effects in the organs under the treatment with NGF/CU after 0, 7 and 14 days. Moreover, the blood routine was also conducted and no changes were observed after 0, 7 and 14 days treatments (Fig. S19b). Overall, these results confirmed the long-term safety and favorable biocompatibility of NGF/CU for *in vivo* applications.

## 3 Conclusion

**Fig. 7:**
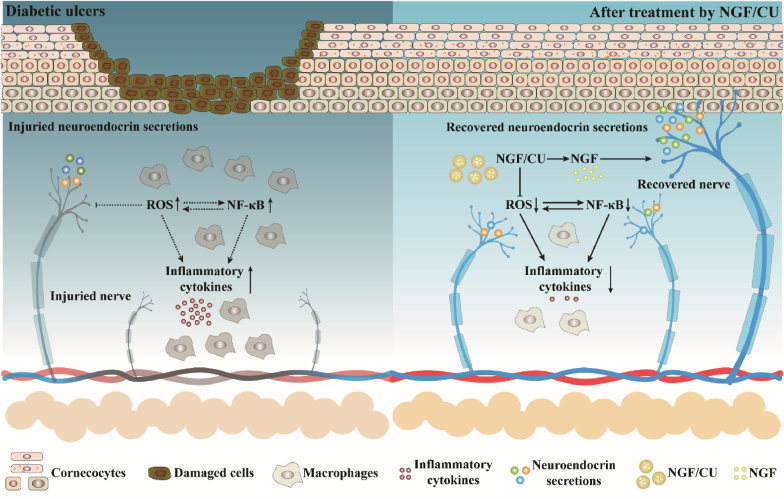
Schematic illustration of mechanism of NGF/CU in the treatment of DUs.

In normal skin wounds, epidermal basal cells (e.g., Keratinocytes, fibroblasts, hair follicle cells, cutaneous nerve cells) tend to migrate to the wound site to promote wound healing [26]. However, the excess accumulation of ROS and diabetic peripheral neuropathy impedes the migration tendency, and the progress of wound healing in DUs is delayed [3], a), b), c), d). Anti-inflammatory therapy has been widely applied for the treatment of DUs but few studies have been focused on repairing peripheral neuropathy. In this study, the NGF/CU containing antioxidants and nerve growth factor in nanoscale MOFs was constructed for DUs treatment. Our results showed that NGF/CU or single CU could effectively scavenge excess ROS and ameliorate the inflammatory microenvironment of DUs. Importantly, NGF/CU could present much better wound healing quality than single CU when the models contained nerve cells. The RNA-seq analysis demonstrated that, besides the ROS-related NF-κB signaling pathway, NGF/CU could promote DUs healing by regulating the skin neuroendocrine system (Fig. 7). Our research demonstrated that effective repair of DUs should not only scavenge reactive oxygen species but also recover the normal regulatory function of cutaneous nerves. Recent progress has revealed the closed connection between nerve system and certain organ injuries. We believe such nerve regulation will become an important strategy for wound healing and other organ injuries.

## Ethics approval and consent to participate

All animal experiments were approved by the Animal Experimentation Ethics Committee of Shanghai Jiao Tong University (A2021124) and were conducted in compliance with all relevant ethical regulations regarding animal testing and research. Patient consent statement: not applicable.

## CRediT authorship contribution statement

**Xiuru Ji:** Writing – review & editing, Writing – original draft, Software, Project administration, Methodology, Investigation, Formal analysis, Data curation, Conceptualization. **Jingwei Zhou:** Software, Project administration, Methodology, Formal analysis, Data curation. **Zengding Zhou:** Project administration, Investigation, Funding acquisition, Formal analysis. **Zeyang Liu:** Methodology, Investigation, Formal analysis, Data curation. **Li Yan:** Methodology, Investigation, Formal analysis, Data curation. **Yuhan Li:** Investigation, Formal analysis, Data curation. **Haiyan Guo:** Methodology, Investigation, Funding acquisition, Data curation. **Weijie Su:** Writing – review & editing, Visualization, Methodology, Investigation, Formal analysis. **Han Wang:** Writing – review & editing, Writing – original draft, Project administration, Methodology, Investigation, Formal analysis, Data curation. **Dalong Ni:** Writing – review & editing, Visualization, Validation, Supervision, Investigation, Funding acquisition, Conceptualization.

## Declaration of competing interest

The authors declare that they have no known competing financial interests or personal relationships that could have appeared to influence the work reported in this paper.
