# Supplementary material for: Recovering skin-nerve interaction by nanoscale metal-organic framework for diabetic ulcers healing
Source: Bioact Mater. 2024 Aug 29;42:112–23. doi: 10.1016/j.bioactmat.2024.08.024 (PMC11402068; doi:10.1016/j.bioactmat.2024.08.024)
Supplement: Multimedia component 1 [file mmc1.docx]

Supporting Information

Recovering Skin-Nerve Interaction by Nanoscale Metal-Organic Framework for Diabetic Ulcers Healing

**Supplementary Materials and Methods**

**Reagents**

N, N-dimethylformamide (DMF), (NH_4_)_2_Ce(NO_3_)_6_, 1,4-benzenedicarboxylic acid (H_2_BDC), and polyvinylpyrrolidone (PVP), were from Adamas. Nerve growth factor (NGF) and lipopolysaccharides (LPS) were obtained from MCE. Phosphate buffered solution (Saline), RPMI 1640, Dulbecco's Modified Eagle Medium (DMEM) High Glucose medium, Penicillin-Streptomycin and fetal bovine serum (FBS) were purchased from Adamas Life. Cell counting kit-8 (CCK-8), DCFH-DA assay kit, DAPI staining kit, GSH and GSSG assay kit, Hydrogen Peroxide assay kit and Total Antioxidant Capacity assay kit were achieved from Beyotime. ELISA kits and inflammatory antibodies were bought from Proteintech.

**Cells and animals**

Human Umbilical Vein Endothelial Cells (HUVECs), Rat pheochromocytoma cells (PC12), and RAW264.7 were obtained from the American Type Culture Collection (ATCC). Human Keratinocytes cells (HaCaTs) and Microglial cells (BV2) were purchased from the National Collection of Authenticated Cell Cultures. Kunming mice and Balb/c mice were approved by the animal care and use committee at Shanghai Jiao Tong University and performed according to the National Institutes of Health Guidelines.

**Experimental apparatus**

The transmission electron microscope (TEM) graph was carried out by FEI Talos F200X. X-ray diffraction (XRD) was measured by Rigaku D/MAX-2250 V. Dynamic light scattering (DLS) was measured by Malvern Zetasizer Nano S. Confocal laser scanning microscopy was carried out on Leica TCS SP8 STED 3X. Cell Cytophagy was detected by Simple Cell-Inductively Coupled Plasma-Mass Spectrometry (SC-ICP-MS, Shimadzu).

**Synthesis of Ce-UiO-66 and NGF/Ce-UiO-66**

4 mmol (NH_4_)_2_Ce(NO_3_)_6_ was dissolved in H_2_O (4 mL) and introduced into a mixed solution consisting of H_2_BDC (4 mmol) and PVP (50 mg) in DMF (30 mL) at a temperature of 100 ℃, followed by stirring for 15 min to obtain Ce-UiO-66 (CU). Subsequently, a solution of NGF at a concentration of 40 μg/mL was added to the dissolved CU and stirred at 25 ℃ for 12 h. After that, the un-loaded NGF was discarded through centrifugation (12000 rpm, 8 min), and the nanomaterials were washed three times with H_2_O, leading to the formation of NGF/Ce-UiO-66 (NGF/CU).

**ROS scavenging in solution**

The content of H_2_O_2_ and total antioxidant capacity were measured by hydrogen peroxide assay kit (Beyotime) and total antioxidant capacity assay kit with ABTS method (Beyotime), respectively. Different concentrations of CU and NGF/CU (0, 6.25, 12.5, 25, 50 ppm) were applied to scavenge H_2_O_2_ (100 μM) as antioxidants. The content of H_2_O_2_ and ABTS was measured by the absorption at 560 nm and 405 nm, respectively.

**Cell viability**

HUVECs and HaCaTs (1 × 10^4^ cells/well ) were seeded in the 96-well plates and cultured at 37 °C. Next, the medium was replaced by the fresh DMEM High Glucose medium with CU or NGF/CU at different concentrations (0, 6.25, 12.5, 25, 50, 100 ppm) and cultured for another 24 h. The medium was discarded and washed with DMEM High Glucose medium. Then 100 μL culture media with 10 μL CCK-8 solution was added and co-incubated with cells for 2 h. At last, the absorbance was measured at 450 nm with a microplate reader. The cell viability of PC12 was cultured with RPMI 1640 medium and detected with the same method.

**Cytophagy**

HUVECs, HaCaTs, and PC12 cells (1 × 10^6^ cells/well) were planted into dishes and cultured at 37℃ for 24 h. Then the cells were incubated with NGF/CU (25 ppm) for 6 h. After incubation, the concentration of Ce in the cells was tested by SC-ICP-MS.

**Detection of ROS in cells**

HUVECs, HaCaTs, and PC12 cells (1 × 10^5^ cells/well) were seeded in confocal dishes and cultured for 24 h. Then the cells were cultured with H_2_O_2_ (100 μM) for 6 h. The cells were further incubated with Saline, NGF, CU, and NGF/CU (25 ppm) for 4 h. After that, DCFH-DA (10 μM) was added to incubate with cells for 0.5 h. Finally, a confocal fluorescence microscope was used for the detection of the fluorescence of DCF.

**Detection of GSSG in cells**

HUVECs, HaCaTs, and PC12 cells (1 × 10^4^ cells/well) were planted into 96-well plates. After 24 h culture, the cells were further cultured with H_2_O_2_ (100 μM) for 6 h. Then the cells were incubated with Saline, NGF, CU, and NGF/CU (25 ppm) for 12 h, respectively. After incubation, the content of GSSG was measured by the GSSG assay kit.

**Detection of TNF-α, IL-6, and IL-10 in cells**

Immunofluorescence staining: Raw264.7 and BV2 cells (1 × 10^5^ cells/well) were seeded in confocal dishes and cultured for 24 h. Then the cells were stimulated by LPS (10 μM) for 8 h. After stimulation, the cells were incubated with Saline, NGF, CU, and NGF/CU (25 ppm) for 4 h, respectively. After that, 4% PFA was used to fix the cells for 15 min, Triton X-100 was used for permeabilizing for 15 min, and 5% bovine serum albumin was used for blocking for 30 min at room temperature. The immunofluorescence of TNF-α, IL-6, and IL-10 in cells was detected by a confocal fluorescence microscope after being cultured with primary antibodies and fluorescent secondary antibodies.

ELISA assay: The cultivation methods were the same as the immunofluorescence staining. After incubation with Saline, NGF, CU, and NGF/CU (25 ppm) for 4 h, the supernatant of the cell culture medium was collected and measured with ELISA assay kits.

**Detection of SP, CORT, and IGF1 in cells**

PC12 cells were seeded in 6-well plates and cultured for 24 h with NGF (10 ng/mL). Then H_2_O_2_ (100 μM) was added into the plates to stimulate the cells for 6 h. After that, the cells were incubated with Saline and NGF/CU (25 ppm) for 4 h, respectively. The supernatant of the cell culture medium was collected and measured with ELISA assay kits. IGF1 was detected using BV2 cells.

***In vitro* wound healing scratch assay**

NGF (10 ng/mL) was incubated with PC12 cells to stimulate nerve functions of PC12 cells. The HUVECs were planted into the lower chambers of transwells and cultured with DMEM High Glucose medium for 24 h. A straight line was scratched with a 200 μL pipet tip in the center of the lower chambers. Then the upper chambers contained 300 μL RPMI 1640 medium and NGF-treated PC12 cells (NGF-PC12) were seeded into part of them. After that, these cells were treated with DMEM High Glucose medium containing Saline, H_2_O_2_, H_2_O_2_ + NGF, H_2_O_2_ (with NGF-PC12 in the upper chamber), H_2_O_2_ + NGF/CU (25 ppm) and H_2_O_2_ +CU (25 ppm, with NGF-PC12 in the upper chamber). After 0 and 24 h treatments, the images of the scratch were collected with a microscope. The closure rate of the gap was calculated via ImageJ software (NIH, Bethesda, MD, USA).

***In vivo* nerve recovery**

Under safe inhalation of isoflurane at 0.41 mL/min and 4 L/min fresh gas flow, the Kunming mice (6 weeks, male) were put on the operating table in the left lateral position. Then a horizontal cut was performed on the skin of the back side of right thigh bone. The blunt separation of the muscle revealed the sciatic nerve completely, subsequently resulting in the creation of a nerve gap measuring approximately 1 cm in length. Afterward, the tibial nerve and common peroneal nerve were identified and ligated with surgical sutures. Then these spared nerve injury (SNI) mice were randomly divided into 5 groups with different treatments through injection in situ. After 14 days of treatment, those mice were sacrificed, and the nerves and muscles were harvested in 4% paraformaldehyde at 24 h. Then these tissues were embedded with paraffin and sliced into 8-μm-thick sections for histology and immunofluorescence analysis. After that, a confocal fluorescence microscope was applied for detection.

***In vivo* DUs healing progress**

BALB/c mice (6 weeks, male) were administered with intraperitoneal injections of STZ (55 mg/kg) daily for 7 days to establish a diabetic mouse model. The blood glucose levels of the mice were observed by employing a glucometer. Mice that exhibited blood glucose levels higher than 16.7 mM were classified as diabetic mice. Then these diabetic mice were anesthetized by isoflurane, and a round incision measuring 8 mm × 8 mm was made on the back using a pair of scissors. These DUs mice were divided into 4 groups and received Saline, NGF, CU (10 mg/kg), and NGF/CU (10 mg/kg) treatments in situ every two days for a week. The wound closure rate and the change of mice weight were collected on days 0, 3, 7, 10, and 14.

**Histological and immunofluorescence analysis**

After 14 days of treatment, those mice were sacrificed, and the healed skins were harvested in 4% paraformaldehyde at 24 h. Then the skin tissues were embedded with paraffin and sliced into 8-μm-thick sections for histological and immunofluorescence analysis. After that, a confocal fluorescence microscope was applied for detection.

**RNA-seq analysis**

Skin slices were obtained from sacrificed DUs mice. In accordance with the manufacturer's recommendations, total RNA was isolated from samples using Trizol Reagent (Invitrogen), and genomic DNA was removed by DNase I. Then, the 2100 Bioanalyser (Agilent) and NanoDrop Technologies were used to assess the RNA's quality. The sequencing library construction consisted of high-quality RNA. Illumina HiSeq X10 (Illumina, Majorbio Bio-pharm Biotechnology Co., Ltd.) was used to complete the isolation of the necessary RNA molecules and reverse transcription of RNA to cDNA, followed by multiplication of primed cDNA molecules and sequencing in accordance with the protocol. The expression of each transcriptome was estimated for bioinformatics using the fragments per kilobase of exon per million mapped reads (FPKM) approach. HTSeq was used to determine the number of reads mapped to each gene. R program was used for the analysis of data (R Foundation for Statistical Computing). The DESeq2 was used to standardize the raw counts, identify differentially expressed genes (fold change 1.5; FDR 0.05), and produce the main component plot. The R package cluster Profiler was used to conduct a gene ontology enrichment analysis, with the differentially expressed genes discovered in the preceding section serving as the input for KEGG enrichment by gene function. The P values were calculated using hypergeometric tests and then submitted to Benjamini-Hochberg correction for multiple testing. The Gene Set Enrichment Analysis (GSEA) was conducted to determine whether the gene sets of interest were significantly enriched under comparable circumstances. Moreover, the Search Tool for the Retrieval of Interacting Genes/Proteins (STRING) method (http://www.string-db.org/) was used to investigate protein-protein interactions between genes. SCytoscape software was used to identify hub genes based on their degrees of connectivity.

**Quantitative reverse transcription PCR**

The skin samples were collected from the sacrificed mice with DUs. The RNA from the skin tissue was isolated using the Trizol reagent (Admas) and converted into cDNA using the Hifair^®^ II 1st Strand cDNA Synthesis Kit (Yeasen). Subsequently, qPCR was performed using the Hieff qPCR SYBR Green Master Mix (Yeasen) on the LightCycler® 480 Instrument II (Roche). The qPCR reaction consisted of 40 cycles, with denaturation at 95 °C for 10 s and annealing at 60 °C for 30 s. The qPCR data was analyzed with a comparative threshold cycle method, where the target amount was normalized to the endogenous reference gene GAPDH in each sample. The primer sequences used were listed below.

GAPDH: Forward ATGGTGAAGGTCGGTGTGAA

Reverse CCCAATACGGCCAAATCCTA

IL-10: Forward GTAGAAGTGATGCCCCAGGC

Reverse CACCTTGGTCTTGGAGCTTATT

TNF-α: Forward TAGCCCACGTCGTAGCAAAC

Reverse GCAGCCTTGTCCCTTGAAGA

IL-1β: Forward TGCCACCTTTTGACAGTGATG

Reverse TGATGTGCTGCTGCGAGATT

IL-6: Forward CTCATTCTGCTCTGGAGCCC

Reverse CAACTGGATGGAAGTCTCTTGC

NF-κB-p65: Forward CAGGGTGACATCACCAAACT

Reverse GGGCTGGCTCTGAGGGA

IκB-α: Forward AACCAGCCAGAAATCGCTGA

Reverse TGCAGACATGTGTGGCCATT

Tac1 (SP): Forward GGTCTGACCGCAAAATCGAAC

Reverse GATCTGGTCACTGTCGGACC

IGF-1: Forward ACAGTGTGTGCCTCCCATAC

Reverse TAGCCTGTGGGCTTGTTGAAG

**Hemolysis analysis**

We used fresh blood from Kunming mice for the hemolysis analysis. Erythrocytes were collected by performing centrifugation at 2000 rpm for 15 min and then washed with Saline three times. After that, the cells were resuspended into Saline (20 mL) for the creation of a 4% suspension. Then the erythrocyte suspension (500 μL) was mixed with 500 μL of a solution containing NGF/CU with concentrations of 25 ppm and 50 ppm, while a 1% Triton-X-100 (TX-100) solution was used as a positive control. The mixtures were then incubated for 2 h at 37°C. Following centrifugation at 4000 rpm for 10 mins, the absorbance of hemoglobin at 450 nm was measured.

***In vivo* biocompatibility assay**

The Kunming mice (4 weeks, male) were given a dose of NGF/CU (10 mg/kg) through intradermal injection. The main tissues (skin, heart, liver, spleen, lung, and kidney) of Kunming mice were dissected for H&E staining at 0, 7, and 14 days. The blood routine of the treated mice was also measured.

**Supplementary Figures**


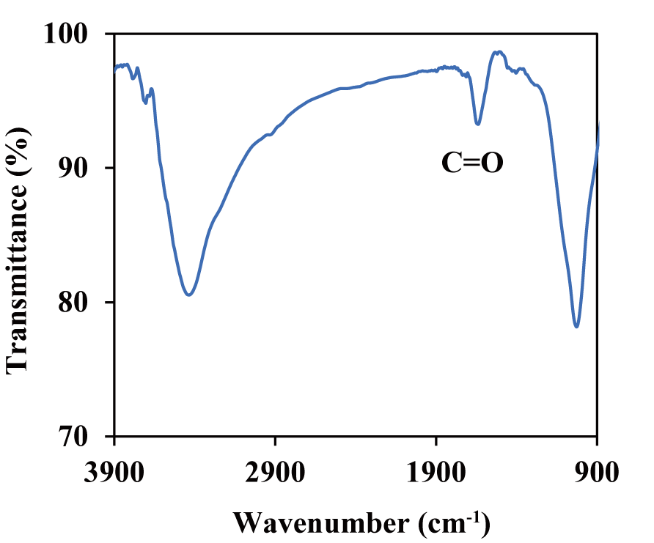


**Figure S1.** Infrared absorption spectrum of CU.


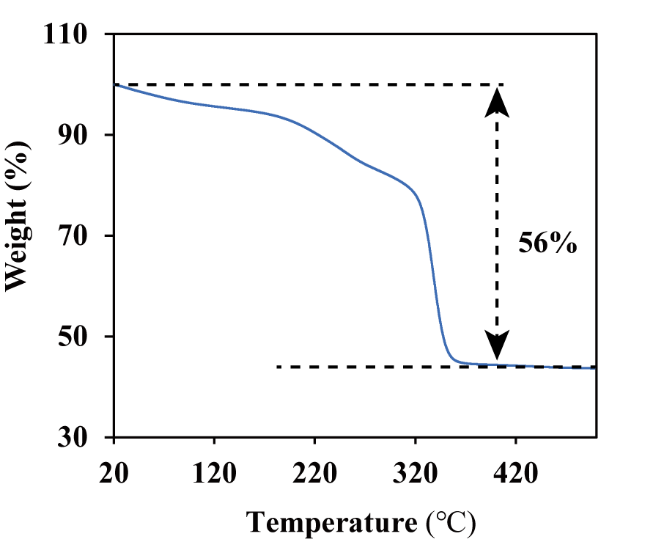


**Figure S2.** Thermogravimetric analysis of CU.


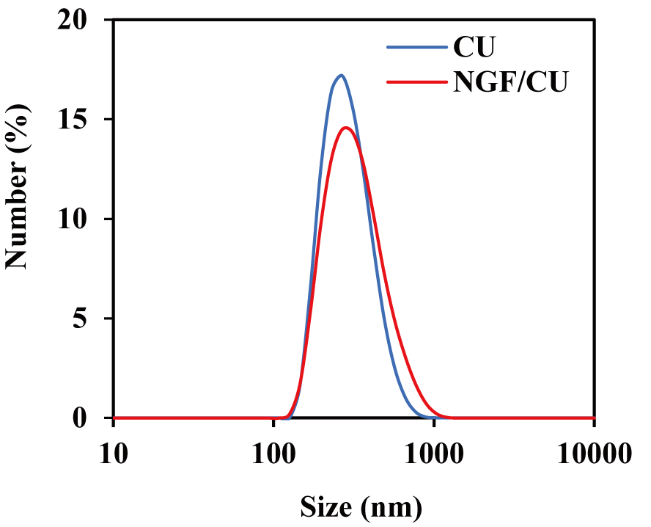


**Figure S3.** The hydrodynamic size of CU and NGF/CU.


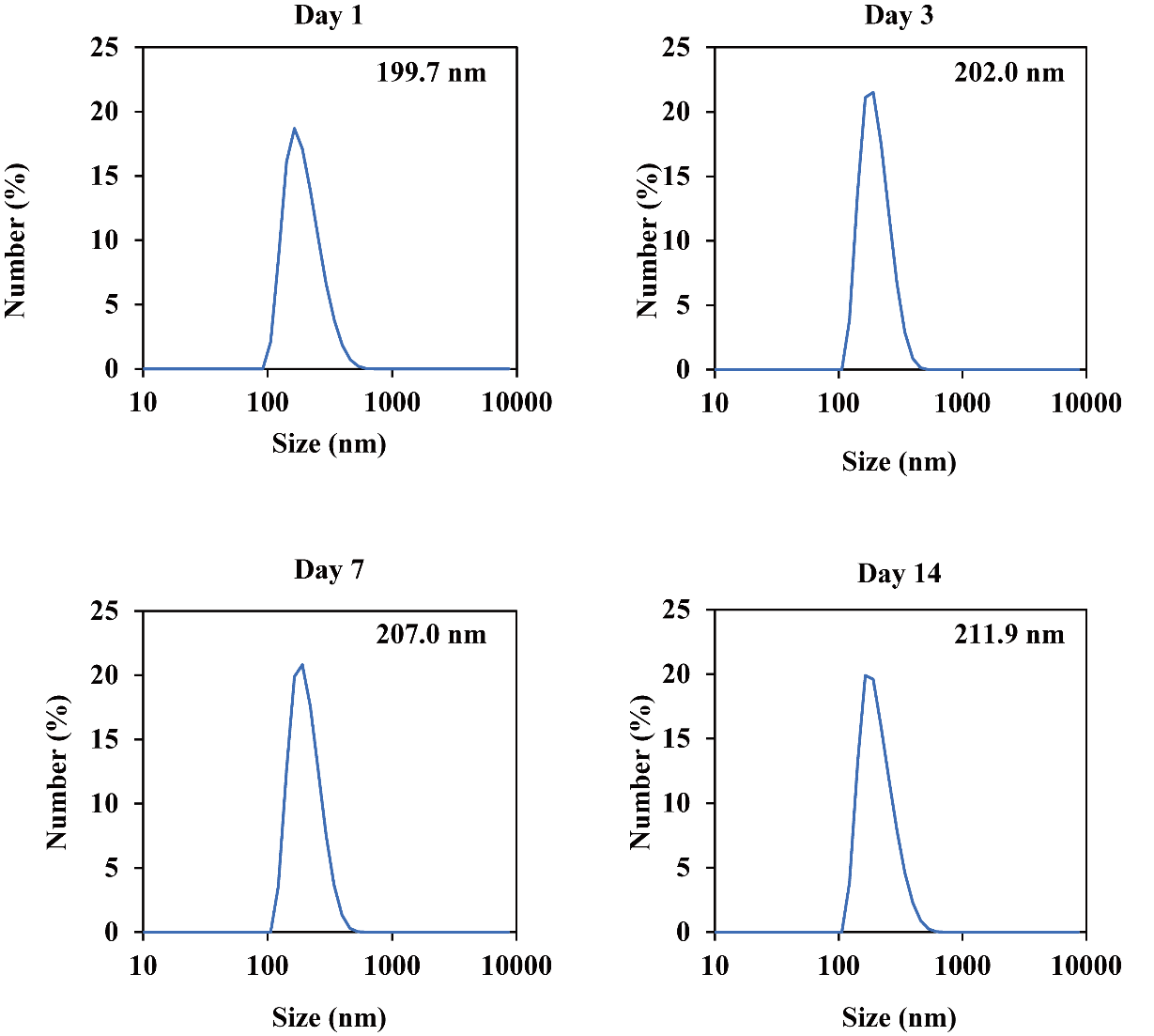


**Figure S4.** The hydrodynamic size of NGF/CU at different time points.


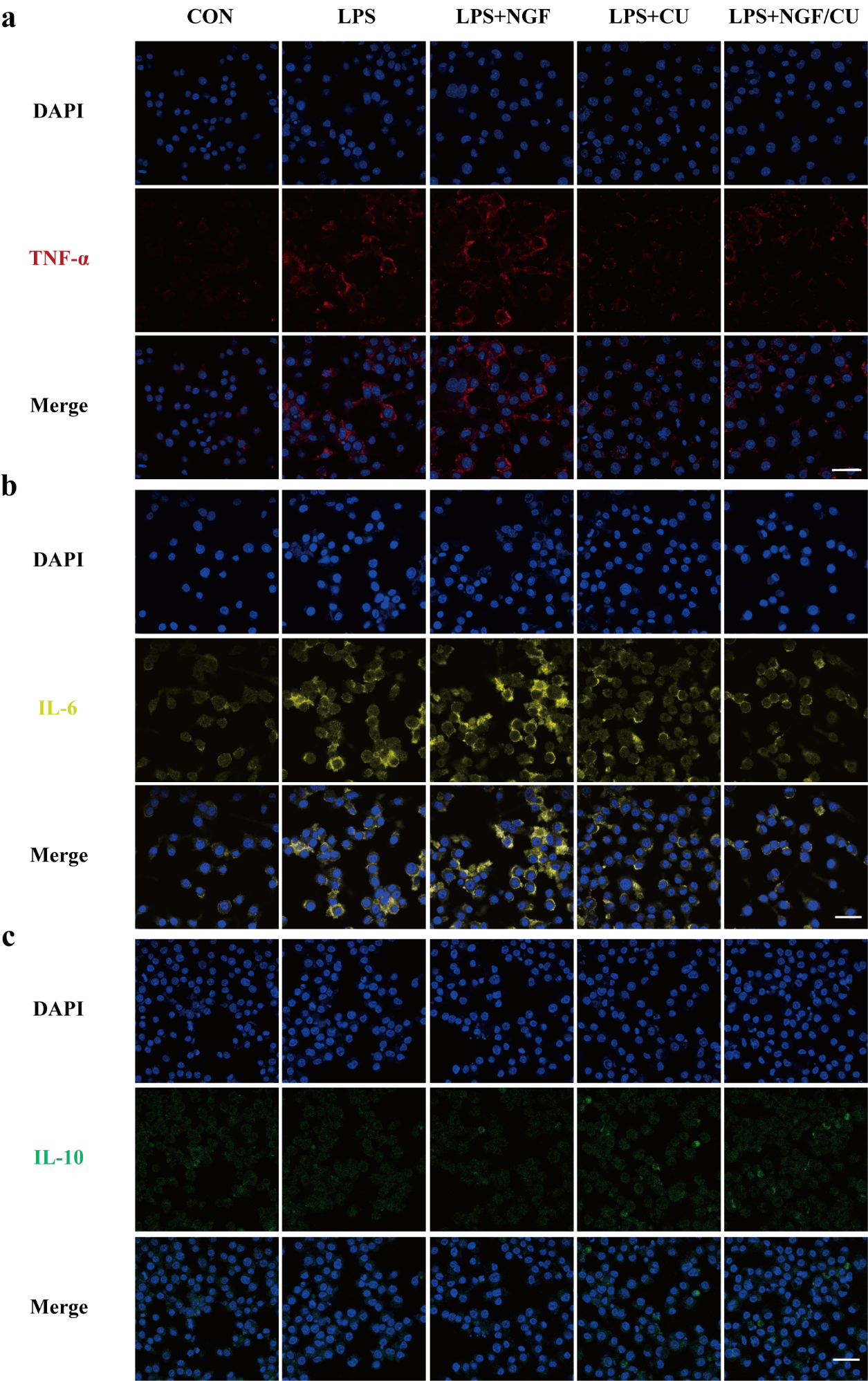


**Figure S5.** Immunofluorescence imaging of (a) TNF-α, (b) IL-6, and (c) IL-10 in RAW264.7 cells. Scale bar: 100 μm.


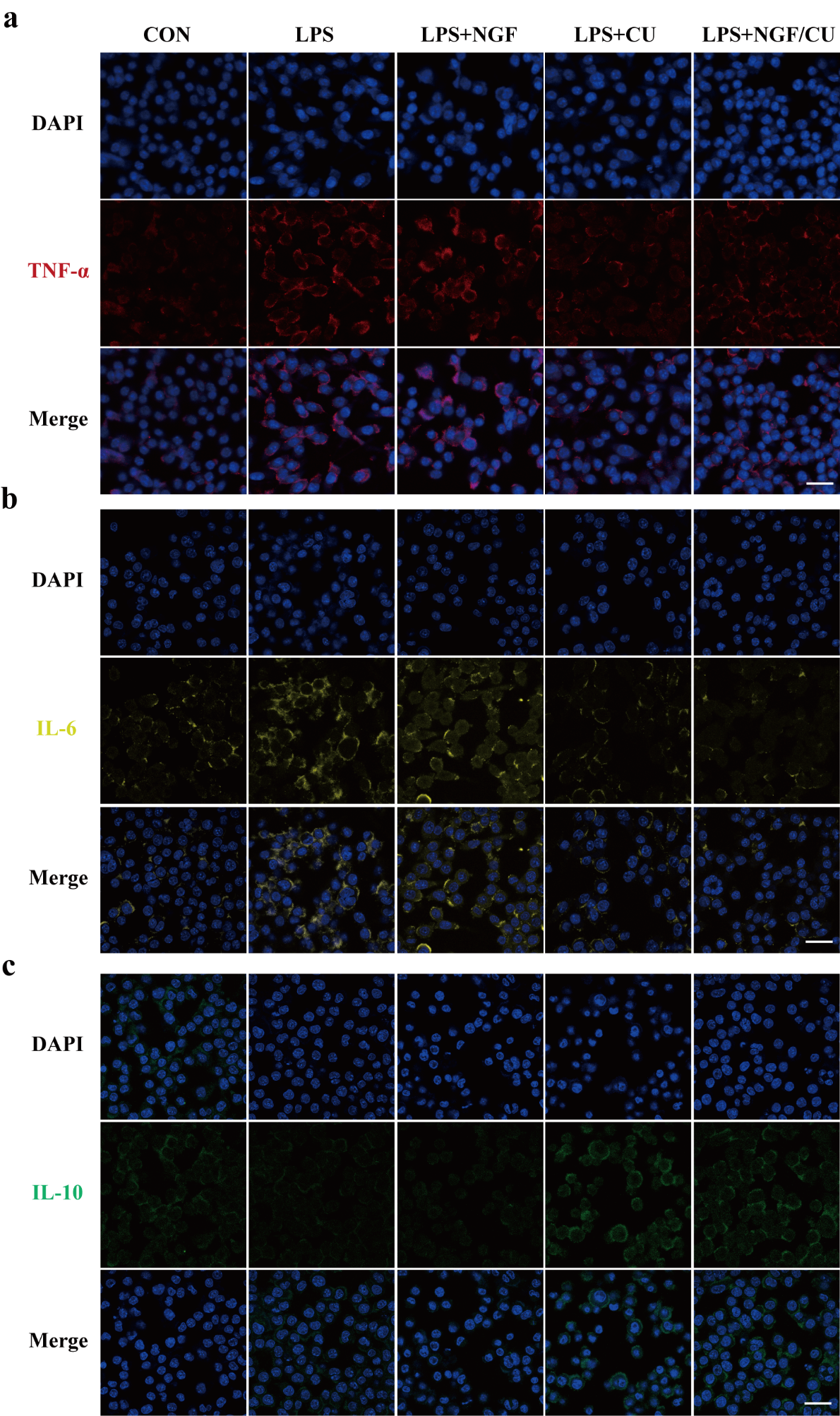


**Figure S6.** Immunofluorescence imaging of (a) TNF-α, (b) IL-6, and (c) IL-10 in BV2 cells. Scale bar: 100 μm.


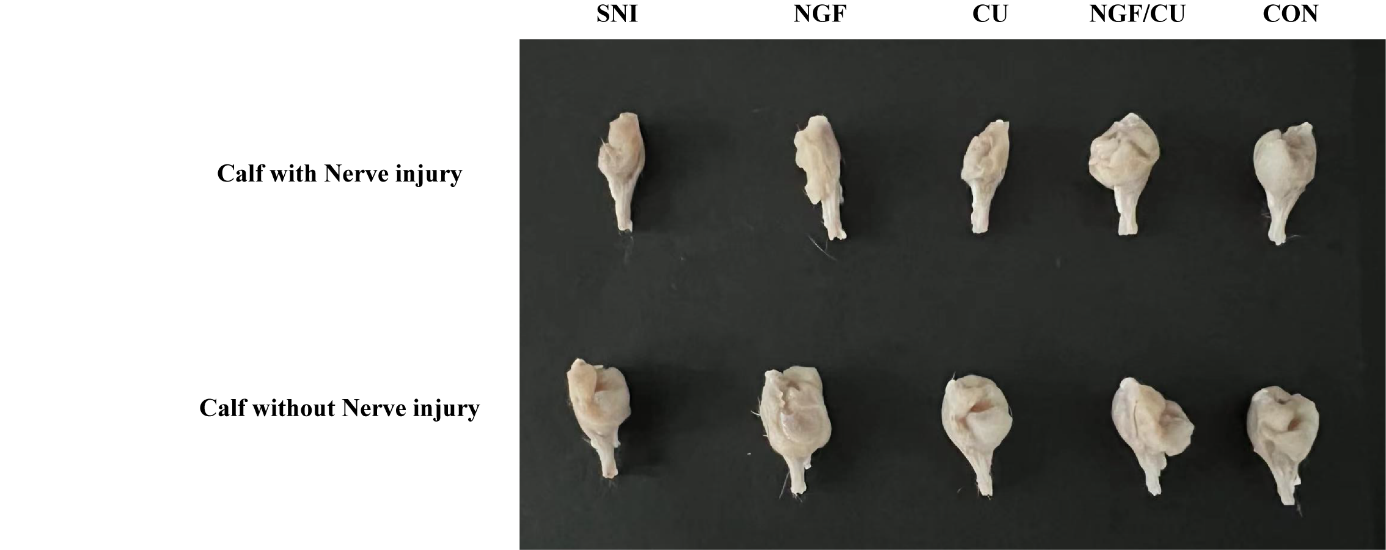


**Figure S7.** Calf of SNI model mice after different treatments.


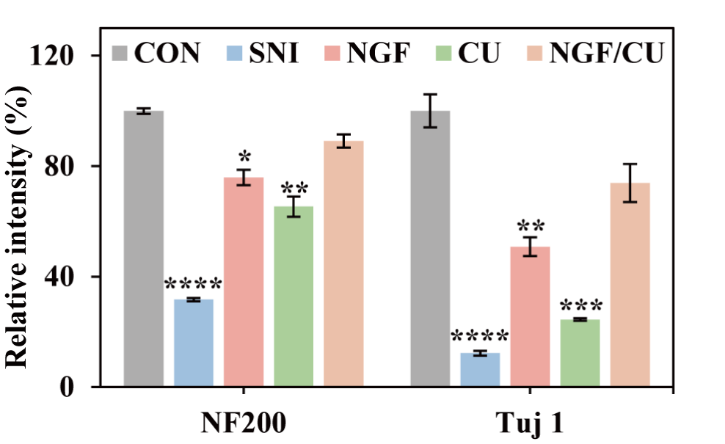


**Figure S8.** Quantitative analysis of NF200 and Tuj1 immunofluorescence intensity for the injured sciatic nerve with different treatments. * indicating 0.01< p < 0.05, ** indicating 0.001< p < 0.01, *** indicating 0.0001< p < 0.001, **** indicating p < 0.0001 according to ANOVA analysis.

**
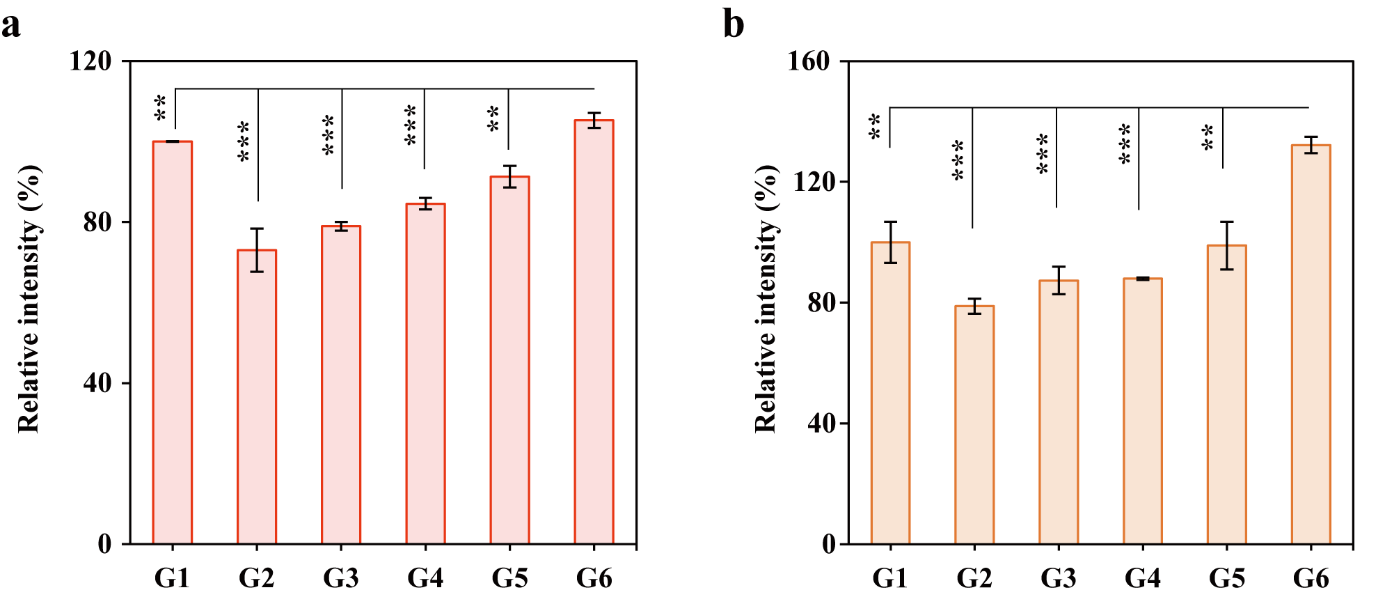
**

**Figure S9.** Quantitative analysis of Ki67 immunofluorescence intensity for (a) HUVECs and (b) HaCaTs. ** indicating 0.001< p < 0.01, *** indicating p < 0.001 according to ANOVA analysis.

**
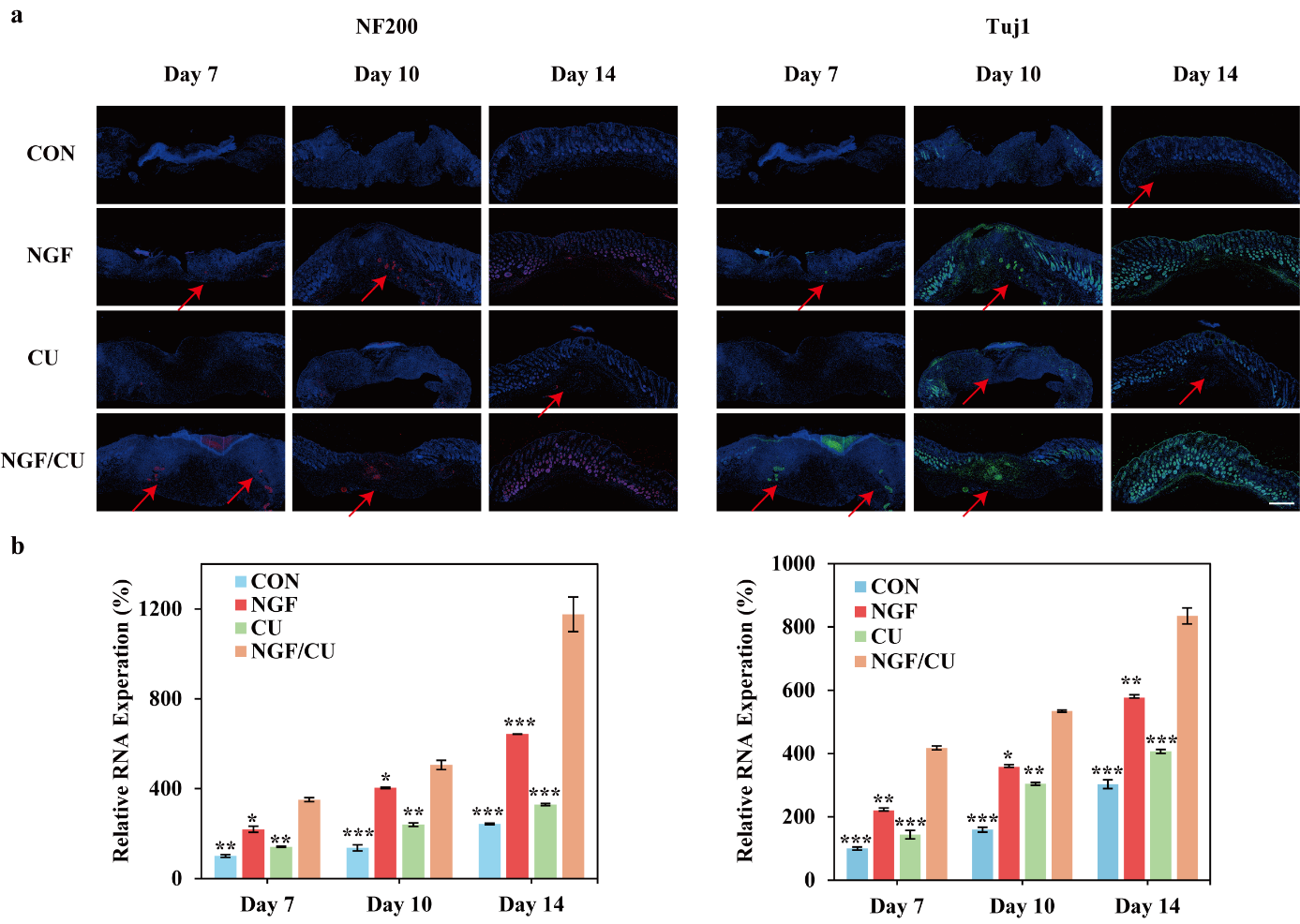
**

**Figure S10.** (a) Immunofluorescence stain of NF200 and Tuj1 in each group at different treatment time points (scale bar: 500 μm). (b) Cutaneous mRNA levels of NF200 and Tuj1 in each group at different treatment time points. * indicating 0.01< p < 0.05, ** indicating 0.001< p < 0.01, *** indicating 0.001< p < 0.0001 according to ANOVA analysis.

**
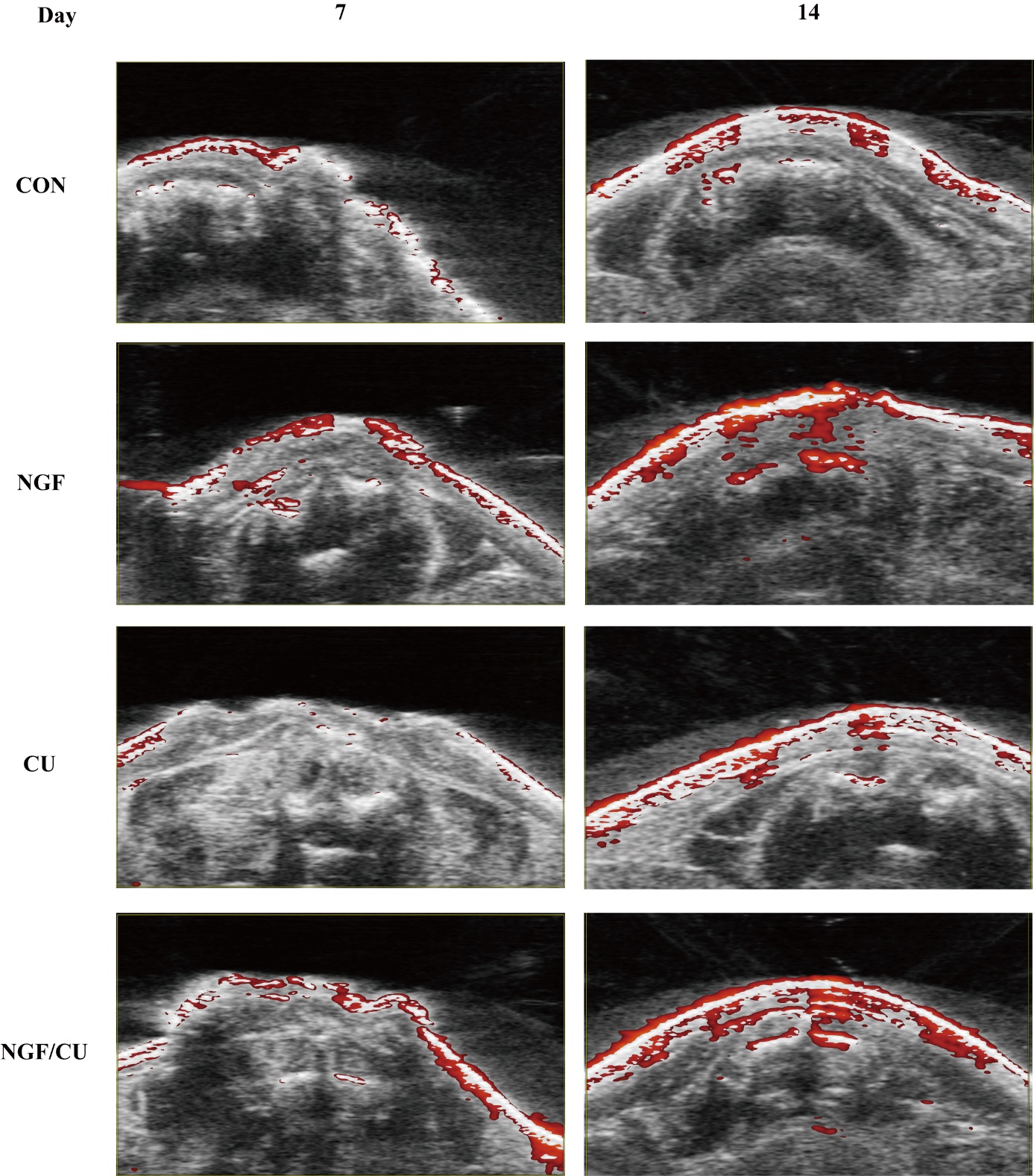
**

**Figure S11.** The blood vessel regeneration of the wounds in each group on days 7 and 14 with ultrasound and photoacoustic imaging systems.

**
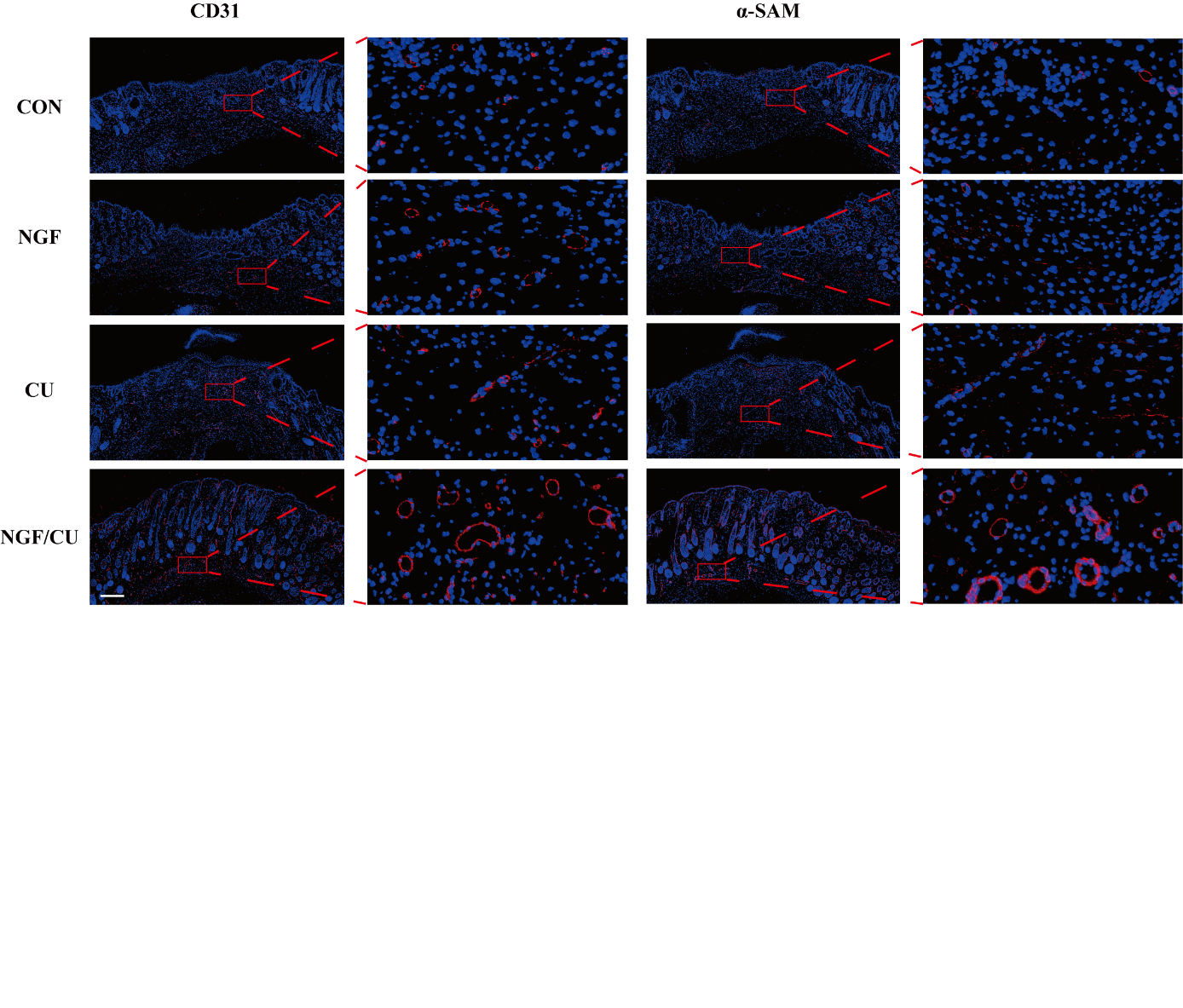
**

**Figure S12.** Immunofluorescence imaging of CD31, and α-SAM with local magnification of the wounds in each group. Scale bar: 500 μm.


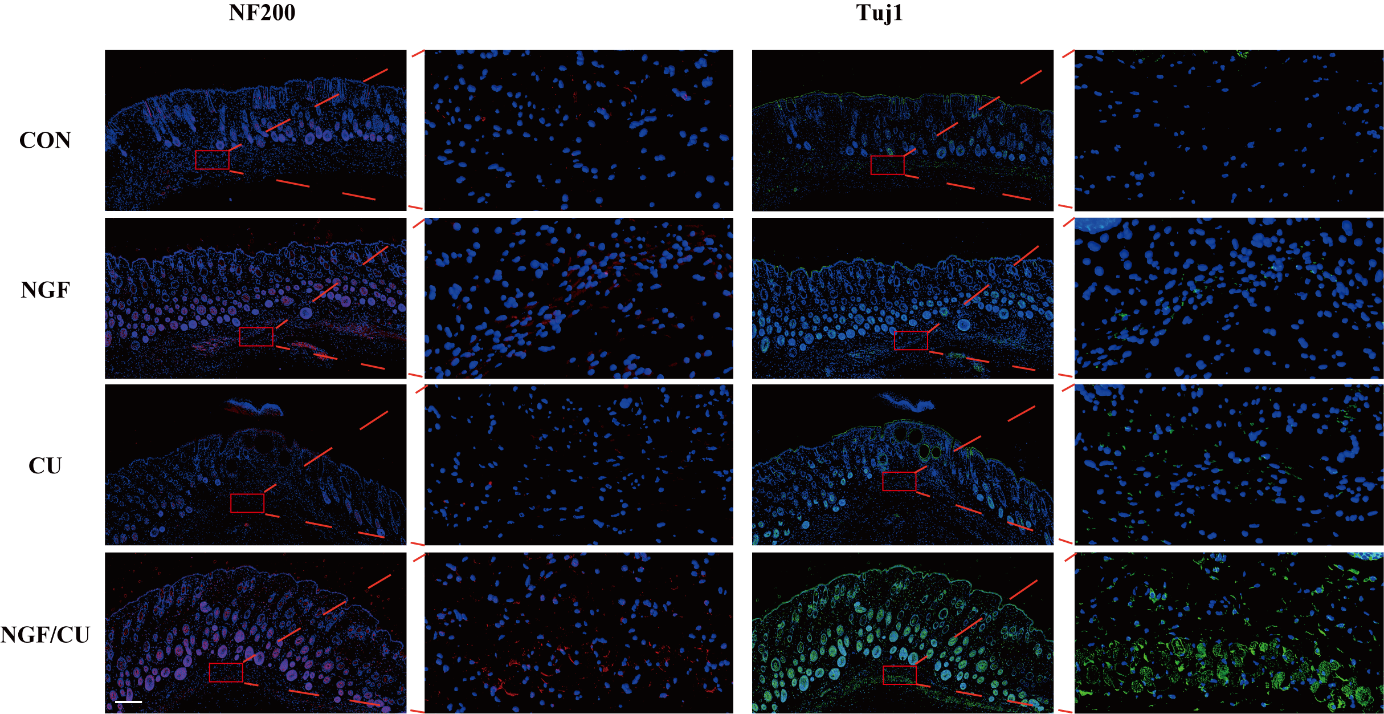


**Figure S13.** Immunofluorescence imaging of NF200, and Tuj 1 with local magnification of the wounds in each group. Scale bar: 500 μm.

**
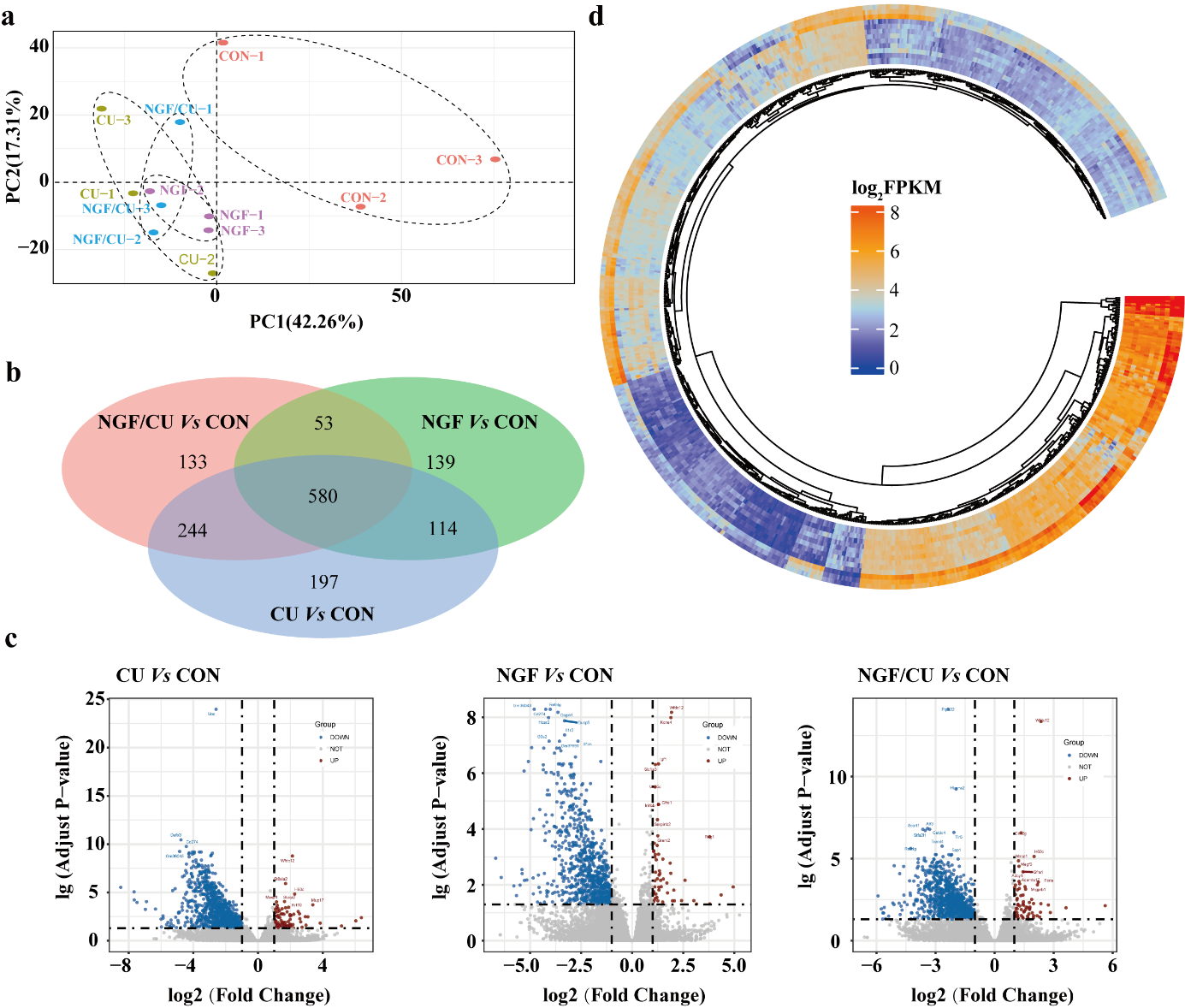
**

**Figure S14.** RNA sequencing analysis of DUs after different treatments. (a) Principal component analysis (PCA) of different groups. (b) Venn diagram of RNA-seq analysis showing different genes in the CU, NGF, and NGF/CU groups compared to the control group. (c) Volcano plot exhibiting the differentially expressed genes (fold change > 1.5; p-adjust < 0.05; upregulated genes: red; downregulated genes: blue) in the CU, NGF, and NGF/CU groups compared to the control group from RNA-seq data, respectively. (d) Heatmap from RNA-seq analysis in the control, NGF, CU, and NGF/CU groups (fold change in the center).


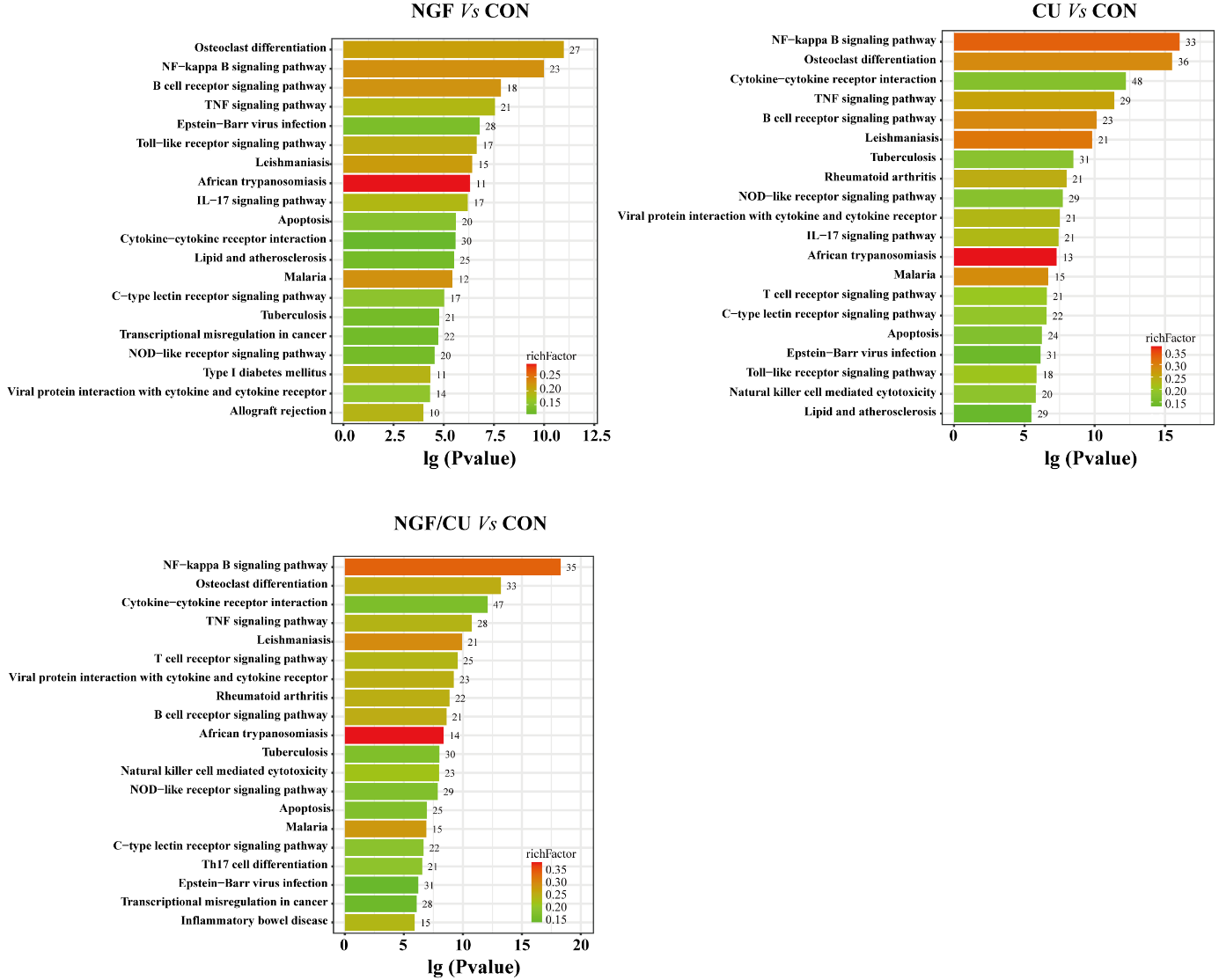


**Figure S15.** The KEGG pathway enriched downregulated genes for DUs with different treatments.


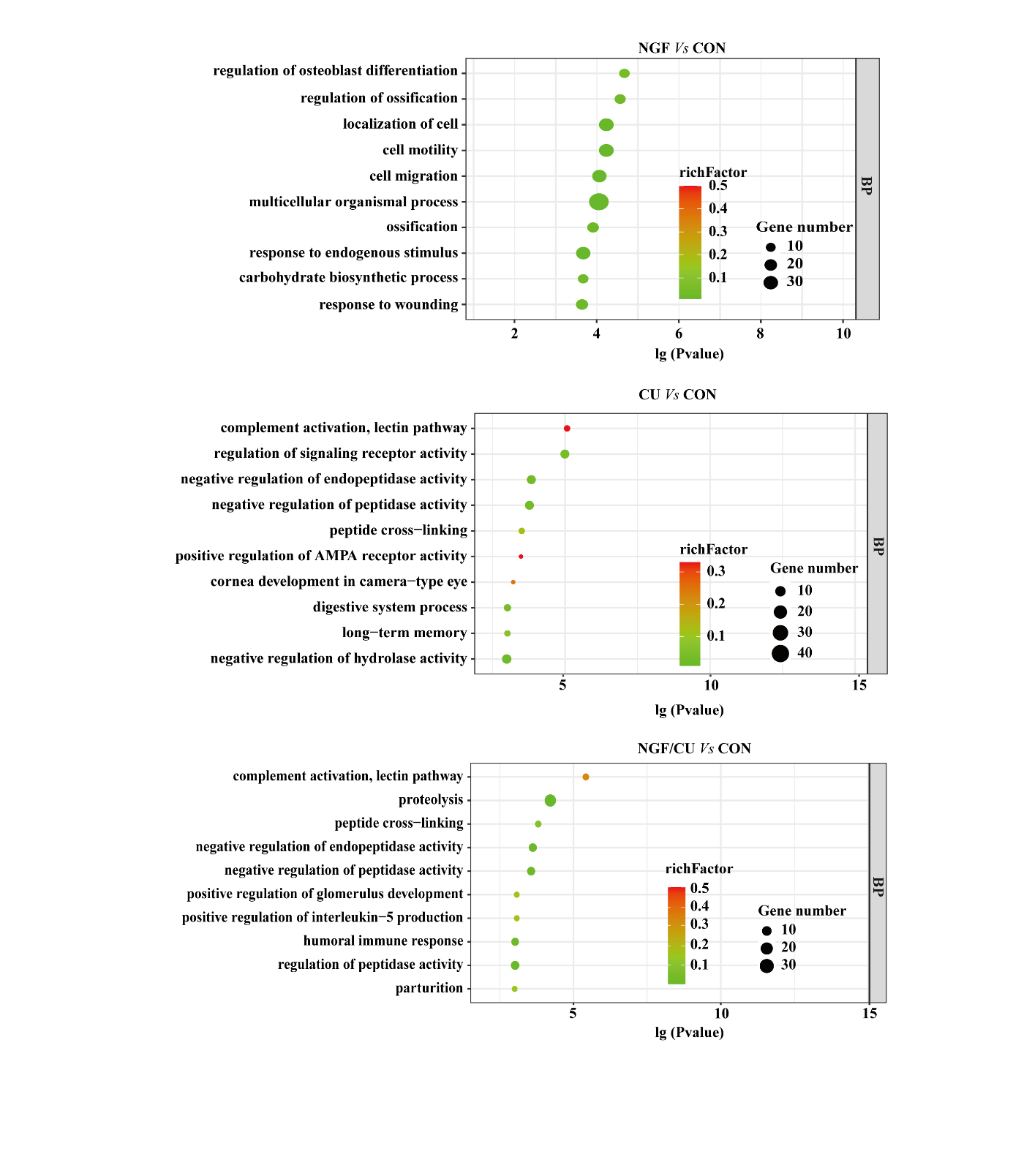


**Figure S16.** The GO pathway enriched upregulated genes for DUs with different treatments.


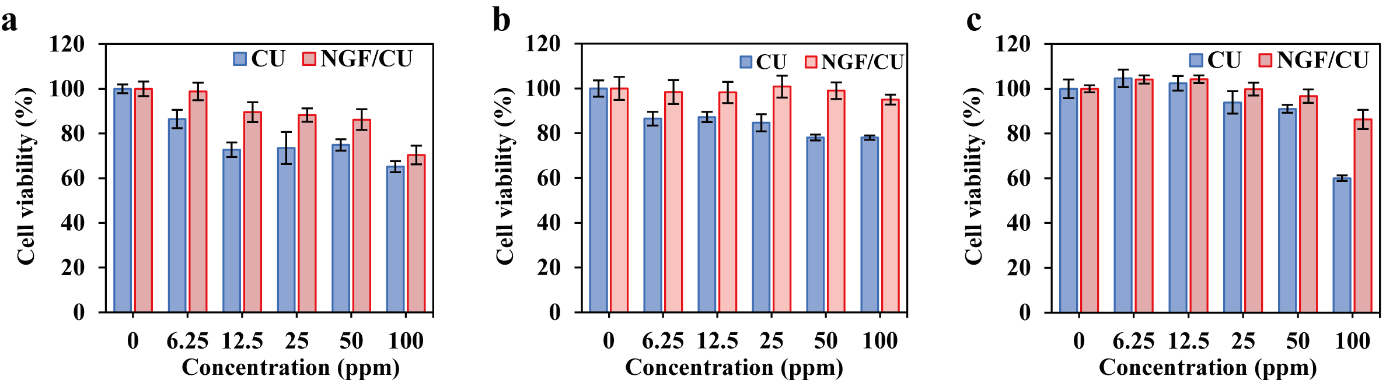


**Figure S17.** Cell viability of (a) HUVECs, (b) HaCaTs, and (c) PC12 cells incubated with CU and NGF/CU for 24 h.


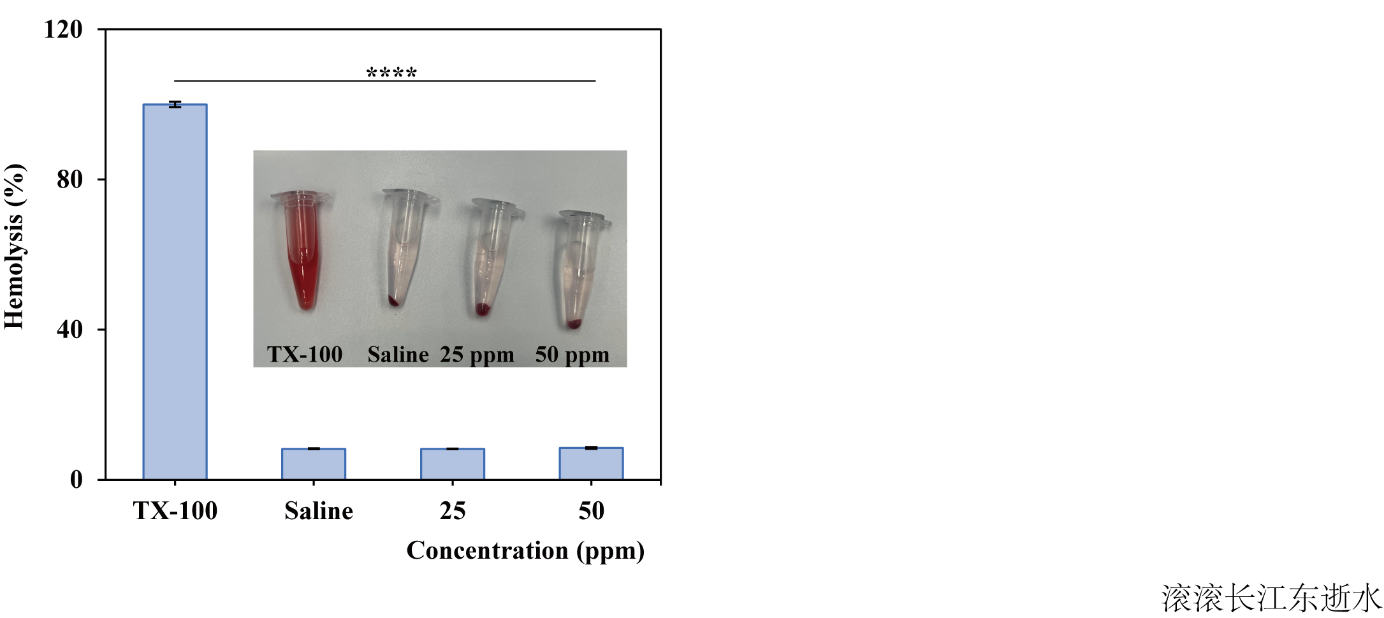


**Figure S18.** The hemolysis analysis of NGF/CU. **** indicating p < 0.0001 according to a Student’s two-tailed t-test.


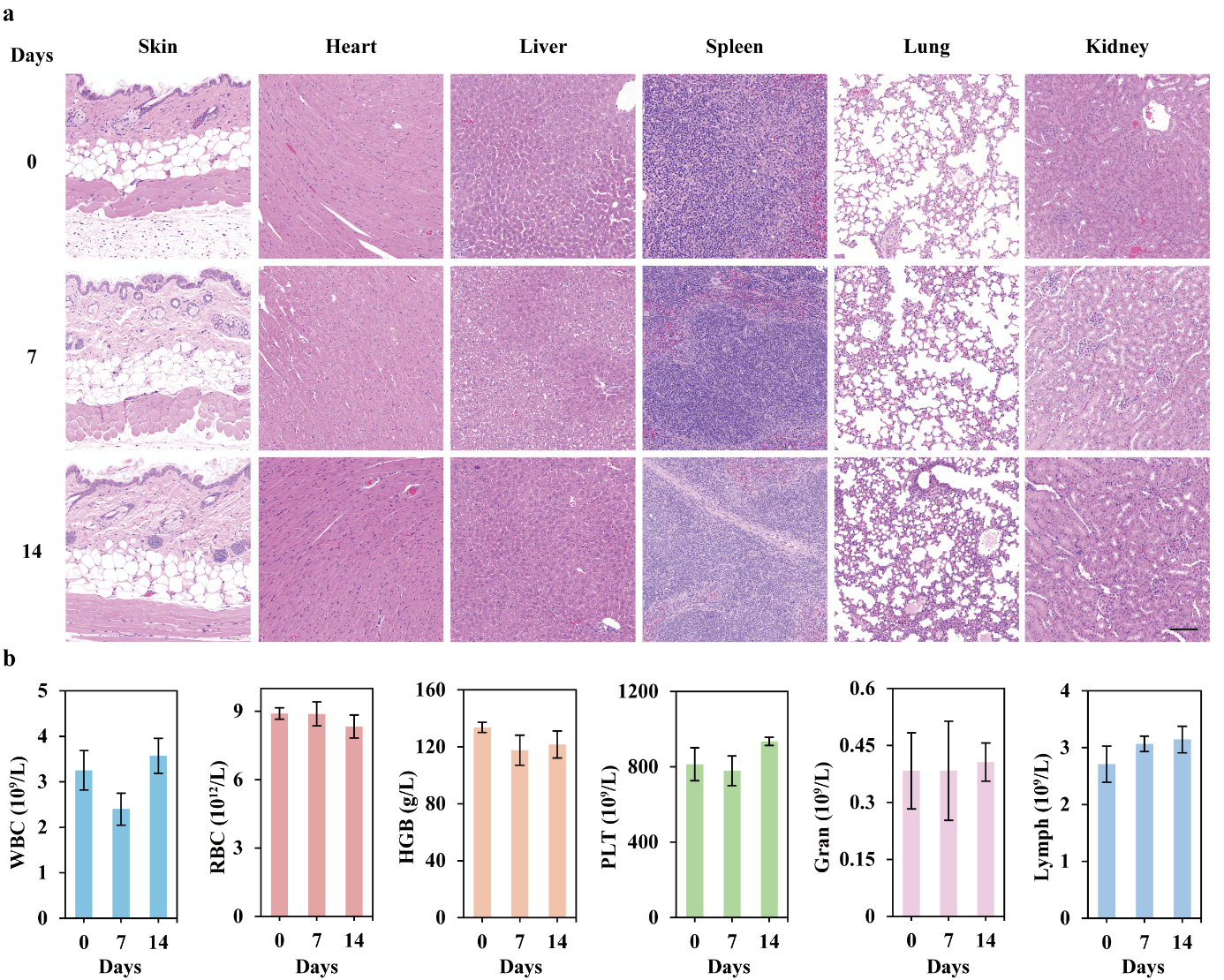


**Figure S19.** (a) H&E staining of major organs (skin, heart, liver, spleen, lung, kidney) after injection of NGF/CU (10 mg/kg) for 0, 7 and 14 days. Scale bar: 100 μm. (b) Blood routine after 0, 7 and 14 days treatments with NGF/CU (WBC: white blood cell; RBC: red blood cell; HGB: hemoglobin; PLT: platelet; Gran: granulocyte; Lymph: lymphocyte).
